# Advances in Naturally and Synthetically Derived Bioactive Sesquiterpenes and Their Derivatives: Applications in Targeting Cancer and Neurodegenerative Diseases

**DOI:** 10.3390/molecules30214302

**Published:** 2025-11-05

**Authors:** Liana R. Cutter, Alexandra R. Ren, Ipsita A. Banerjee

**Affiliations:** Department of Chemistry and Biochemistry, Fordham University, 441 East Fordham Road, Bronx, New York, NY 10458, USAaren6@fordham.edu (A.R.R.)

**Keywords:** sesquiterpenes, sesquiterpene quinones, sesquiterpene lactones, nanotherapeutics, conjugates

## Abstract

Sesquiterpenes are a diverse class of natural products that have garnered considerable interest for their potent bioactivity and structural variability. This review highlights advances in the derivatization of various sesquiterpene lactones, quinones, and alcohols, particularly in targeting cancer and neurodegenerative diseases. The structural modifications discussed include the incorporation of triazole, arylidene, or thiol moieties with eudesmane, guaiane, and germacranolide-type sesquiterpenes, among others. In addition, the conjugation with chemotherapeutics, as well as the development of nanoscale therapeutics, is also discussed. Such modifications have expanded the pharmacological potential, enabling improved specificity, cytotoxicity profiles, and sensitization toward tumor cells. Additionally, sesquiterpenes such as parthenolide (**20**), pterosinsade A (**176**), and cedrol (**186**) have demonstrated potential in mitigating neurodegeneration via anti-inflammatory and antioxidant signaling pathway-modulation mechanisms, with potential applications in Alzheimer’s, Parkinson’s, and ALS diseases. Mechanistic insights into redox signaling modulation, NF-κB inhibition, ROS regulation, and disruption of aggregation underscore their multifaceted modes of action. This review highlights the translational promise of sesquiterpene derivatives as dual-action agents for potential drug development in a plethora of diseases that are caused by inflammation and free-radical damage. It provides a framework for future rational design of multifunctional drug candidates and therapeutics.

## 1. Introduction

Terpenes are a class of bioactive compounds derived from natural resources that contain isoprene units and have been known for centuries for possessing a plethora of biological and pharmacological applications [1]. While chemically, terpenes are generally hydrocarbons, terpenoids are a class of compounds that include other functional groups, including heterocyclic moieties [2]. Among these are a vibrant class of compounds known as sesquiterpenes, which contain three isoprene units and are generally considered one of the largest groups of secondary metabolites obtained from plants and marine organisms [3,4]. They can be classified as linear, branched, or cyclic. Biosynthetically, it has been shown that sesquiterpene synthases are a class of metalloenzymes that play a critical role in the synthesis of sesquiterpenes by initiating the cleavage of farnesyl diphosphate, which allows for the removal of diphosphate (PPi) and the generation of carbocation intermediates, which are then involved in a series of cyclization reactions, eventually leading to the formation of a variety of sesquiterpenes [5]. Over the years, several sesquiterpenes and their derivatives have been isolated, purified, and extracted from roots, leaves, plant essential oils, marine organisms, or mycelia for their medicinal properties. Some of the plant families, such as Lamiaceae, Asteracea, Rutaceae, Schisandraceae, Zingiberaceae, and Myrtaceae, are rich in sesquiterpenes. In addition, sesquiterpenes are also extracted from *Citrus aurantium*, *Artemisia annua*, *Geranium macrorrhizum*, *Achillea vermacularis*, and *Helenium amarum*, among others [6,7,8]. For example, the sesquiterpene alcohol farnesol, derived from essential oils such as neroli and citronella, is known for its anticancer properties against colon cancer and has also demonstrated strong anti-inflammatory activity [9]. Sesquiterpene lactones, such as santamarin (**1**) (isolated from *Saussurea lappa*) and onopordopicrin (**2**) (isolated from *Articum lappa*), have also shown strong potential as antioxidants [10,11]. In a recent study, Singh and co-workers elucidated the chemical components of davana essential oil (DO). Their results revealed the presence of sesquiterpenes with varying functionalities, such as bicyclogermacrene (**3**), *cis* davanone (**4**), *cis* hydroxy davanone (**5**), spathulenol (**6**), and *trans* davanone (**7**), along with other components such as *trans*-ethyl cinnamate. Furthermore, both DO and *cis* davanone exhibited strong anti-inflammatory properties, as was proven by the inhibition of production of tumor necrosis factor-α (TNF-α) and interleukin-6 in primary macrophage cells that had been treated with lipopolysaccharide [12]. In another interesting study, essential oil (EO) derived from *Cordia africana* was shown to contain eighty compounds of interest. Among those were bicyclic sesquiterpenes, such as β-caryophyllene (**8**) and germacrene D (**9**), as well as δ-cadinene (**10**). Furthermore, the oil was found to be cytotoxic toward MCF-7 breast cancer cells and induced apoptosis [13]. In a study conducted by Calvopiña and co-workers, a novel sesquiterpene essential oil derived from the Andean species *Jungia rugosa* was found to be composed exclusively of sesquiterpenes. Specifically, the authors found that the major components included γ-curcumene (**11**) and β-sesquiphellandrene (**12**), along with α-copaene (**13**), β-bisabolene (**14**), and α-*cis* bergamotene (**15**), among others, as minor components. Overall, the authors identified over 55 sesquiterpenes in the EO. Additionally, the EO showed weak inhibitory activity against the enzyme acetylcholinesterase in vitro [14]. These compounds can be further developed through strategic chemical functionalization into more potent AChE inhibitors for treatment against neurodegenerative diseases. To further enhance the potency of plant-derived EOs, various nanoformulations have also been developed. An extensive review of plant-derived EO nanoformulations against breast cancer was recently published by Thalappil and co-workers [15].

Thus, numerous sesquiterpenes of plant origin have shown high bioactivity and have therefore been investigated for their clinical potential. However, the broad therapeutic use of sesquiterpenes obtained from EOs has been somewhat limited to the use of crude extracts due to synthetic impediments and limited information about the structural requirements for selectivity with respect to a desired therapeutic activity. Over the years, there has been a concerted effort for the rational synthesis of many of these sesquiterpenes and their derivatives with better yields, higher purity, and stereochemical selectivity for wider use in medicine. For example, the total synthesis of the tricyclic sesquiterpene (-)-merrillianin (**16**) was recently reported by Shiina and co-workers [16]. This compound has been touted as applicable in the potential treatment of rheumatoid arthritis as well as in neurological diseases. The total synthesis of (-)-cucumin H (**17**), a triquinane-based sesquiterpene commonly isolated from fungi, has also been reported [17]. It was shown to display anti-inflammatory and antimicrobial activity as well as cytotoxicity against tumor cells [18]. Among the various derivatives of sesquiterpenes, sesquiterpene lactones (SLs) have gained prominence due to their numerous therapeutic benefits. Sesquiterpene lactones are biosynthesized by oxidation and hydroxylation processes of various sesquiterpenes by cytochrome P450 enzymes [19]. While Grieco and co-workers successfully demonstrated the total synthesis of sesquiterpene lactones (±)-vernolepin (**18**) and (±)-vernomenin (**19**) in their pioneering work decades ago [20], over the years, several SLs have been successfully synthesized [21,22,23,24]. SL-derived drugs, such as parthenolide (PTL) (**20**) (an inhibitor of the NF-κB signaling pathway that induces apoptosis in cancer stem cells), thapsigargin (**21**) (a potent sarcoplasmic/endoplasmic reticulum Ca^+2^ ATPase (SERCA) pump inhibitor), and artemisinin (**22**), a cadinanolide with anti-inflammatory, antimicrobial, and anti-malarial properties, are now being explored as anticancer drugs [25,26,27].

In this review, we will be focusing on the derivatives of sesquiterpenes, with particular emphasis on synthetic derivatives, for cancer therapeutics and their drug conjugates developed particularly over the past fifteen years, including recently published work (2025). Progress in nanotherapeutics that may have potential applications, particularly in cancer drug development and in mitigating neurodegenerative diseases, is also discussed.

## 2. Antitumor Activity of Sesquiterpene Lactone Derivatives

### 2.1. Naturally Derived Sesquiterpenes

**Figure 1:**
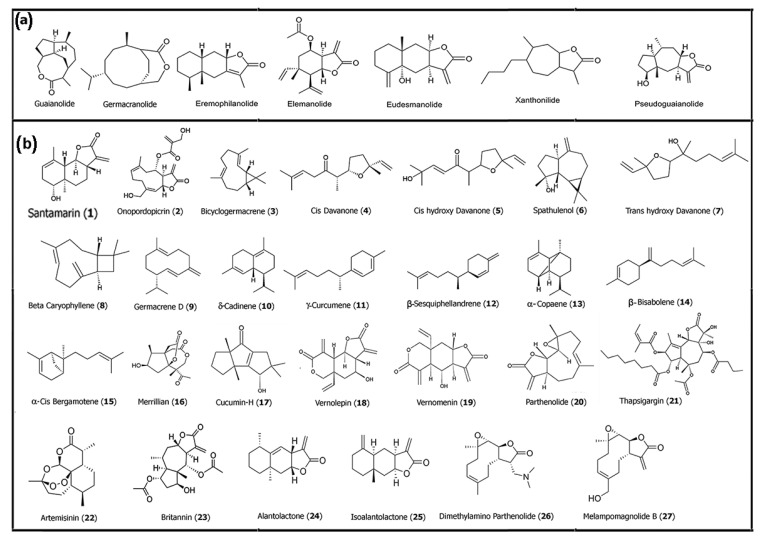
(**a**) Core structures of common naturally derived sesquiterpene lactones. (**b**) Structures of some sesquiterpenes and SLs that have successfully been utilized for medicinal purposes. Structures were drawn using Chem Draw 23.1.

There have been numerous review articles in the past that have provided in-depth information about naturally derived sesquiterpene lactones and their therapeutic applications [28,29,30]. In particular, the anticancer properties of SLs are largely attributed to the electrophilic α-methylene-γ-lactone moiety, which readily undergoes Michael-type addition with nucleophilic thiol groups present in key cysteine residues of intracellular proteins such as IκB kinase (IKK), which is essential for NF-κB activation, and Kelch-like ECH-associated protein 1 (Keap1), which modulates Nrf2 stability [31]. This covalent interaction can result in irreversible inhibition of signaling proteins that are critical for cancer cell survival and inflammation. One such SL, britannin (**23**), a pseudoguaianolide-type SL isolated from *Inula britannica*, has gained recognition for its ability to modulate multiple redox-sensitive pathways simultaneously. In a detailed review of its pharmacological actions, Bailly reported that britannin (**23**) can inhibit major signaling axes, including nuclear factor-κB (NF-κB), signal transducer activator of transcription 3 (STAT3), and nuclear factor erythroid 2-related factor 2 (Nrf2) pathways. Through inhibition of NF-κB signaling, it reduces transcription of anti-apoptotic and inflammatory genes, such as Bcl-2, survivin, and interleukins, thereby promoting apoptosis in various cancer cell types. Interestingly, as a suppressor of Nrf2, britannin (**23**) acts as a master regulator of the antioxidant response, sensitizing tumor cells to oxidative damage and chemotherapeutic stressors [32]. While britannin (**23**) is one of the most studied pseudoguaianolides, guaianolides such as alantolactone (**24**) and isoalantolactone (**25**) have demonstrated comparable mechanisms. For example, Vu and colleagues showed that SLs such as alantolactone (**24**) bearing an α-methylene-γ-lactone moiety promoted the ectodomain shedding of TNF-α receptor 1 (TNFα-R1) and inhibited NF-κB activation in vitro by modulating IKK activity, leading to the suppression of pro-inflammatory gene expression [33]. The chemical structures of some of the common sesquiterpene families and examples of some of the sesquiterpenes and SLs discussed are shown in Figure 1.

**Figure 2:**
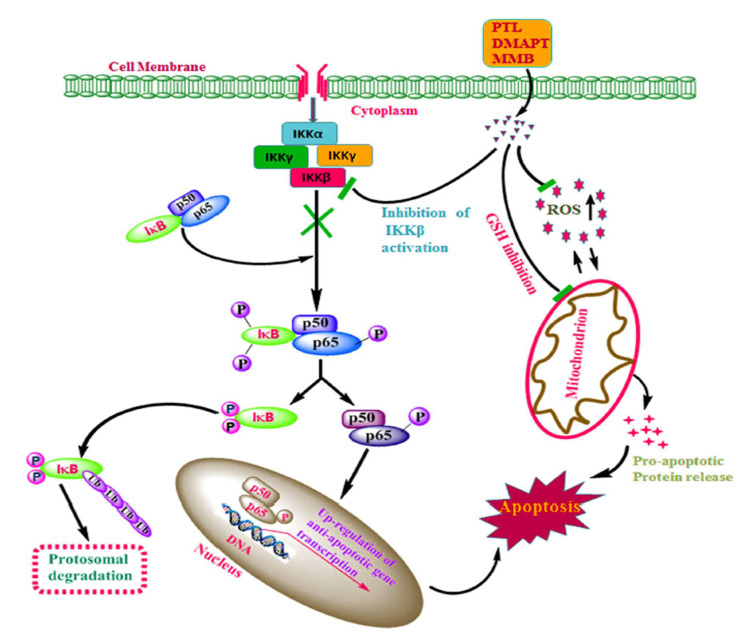
Predicted mechanisms of sesquiterpene lactones (SLs) such as parthenolide (PTL), DMAPT, and melampomagnolide B (MMB) that involve inhibition of IKKβ activation, leading to reduced phosphorylation of IκB and/or p65, which downregulates antiapoptotic gene transcription and sensitizes tumor cells to apoptosis. SLs also inhibit the mitochondrial glutathione system, leading to higher ROS and oxidative stress, further promoting apoptosis in tumor cells [Reproduced with permission from Elsevier [34].

These findings support a broader mechanistic paradigm where structurally conserved electrophilic moieties in SLs exert anticancer effects by covalently modifying thiol-rich regulatory nodes in oncogenic signaling cascades. The general mechanisms of action of SLs, such as PTL (**20**), DMAPT (dimethylamino parthenolide) (**26**), and melampomagnolide B (**27**), toward targeting tumor cells are summarized in Figure 2.

Sesquiterpene lactones have also been shown to be potential inhibitors of epidermal growth factor receptor (EGFR) and vascular endothelial growth factor receptors (VEGFRs), both of which have been found to be over-expressed in many cancers and are therefore targets for the development of new antitumor drugs [35]. For example, in a recent study, Nerdy and co-workers demonstrated through molecular docking studies that SLs, such as vernodalol (**28**), vernodalin (**29**), vernolepin (**18**), vernolide (**30**), vernomygdin (**31**), ferutinin (**32**) (an ester), and hydroxyvernolide (**33**), showed higher binding and better inhibition toward these receptors compared to the known anticancer drug cyclophosphamide [36]. A summary of selected sesquiterpene SLs (**20**, **23**, **24**, **25**, **28**, **29**, **34**, **35**, **36**, and **37**) and the sesquiterpene ester ferutinin (**32**) [37,38,39,40,41,42,43,44,45,46,47] and their effects on multiple tumor cell lines is shown in Table 1. The chemical structures of compounds (**28**) through (**37**) are shown in Supplementary Information Figure S1.

### 2.2. Remodeling of Sesquiterpene Extracts via In Situ Chemical Derivatization

To overcome the limitations of natural product isolation and scaffold rigidity, Adessi and co-workers recently developed a diversity-enhanced extracts (DEE) platform that employs in situ chemical derivatization of crude plant extracts to generate novel bioactive analogues. Applying this strategy to *Ambrosia tenuifolia extract*, which is rich in sesquiterpene dilactones psilostachyin A (Psi A) (**38**), psilostachyin B (Psi B) (**39**) (structure shown in Supplementary Information Figure S2), and psilostachyin C (Psi C) (**40**), the authors were able to chemically modify the extract using a series of selective extractions and derivatization directly on the extract matrix [48]. They utilized a ring distortion model, which allowed them to obtain new compounds in which the ring structure was altered in addition to the known bioactive compounds.

**Scheme 1:**
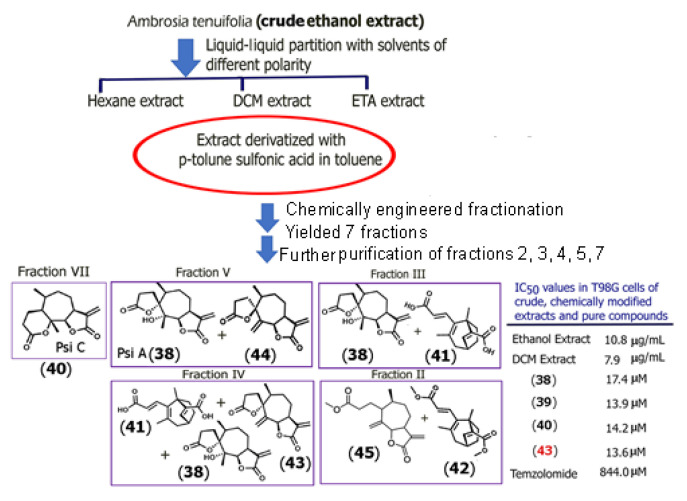
Scheme showing the diversity-enhanced extraction approach, which employs multiple extractions, in situ chemical reactions, and fractionations to obtain novel bioactive compounds as well as known compounds from crude plant extracts. A total of eleven new compounds were obtained, of which five are shown (compounds **41**, **42**, **43**, **44,** and **45**) in addition to Psi A and Psi C. The IC_50_ values were obtained based on MTT cytotoxicity assays conducted in glioblastoma T98G cells. Only those compounds with IC50 < 100 μM were further studied. Adapted from [48]).

To begin, the crude ethanolic extract of *Ambrosia tenuifolia* was prepared and was then partitioned with different solvents of varying polarities (hexane, dichloromethane (DCM), and ethyl acetate). The DCM extract was subjected to chemical reactions with p-toluene sulfonic acid and was then further subjected to fractionation. The cytotoxic activity of the extracts at each stage toward T98G glioblastoma cells was examined. A total of eleven new compounds were obtained, of which five are shown in Scheme 1, which illustrates the methodology used. Among the new compounds (**41–45**), which included epimeric derivatives of Psi A (compounds **43** and **44**), compound **43** showed a lower IC_50_ value than those obtained for the known sesquiterpene dilactones Psi A, B, or Psi C against glioblastoma, T98G cells. All extracts showed remarkably lower IC_50_ values compared to the current chemotherapeutic drug for glioblastoma (temozolomide). This method represents a paradigm shift by integrating synthetic chemistry with extract-based drug discovery to yield non-natural sesquiterpene derivatives with medicinal relevance, bypassing the need for de novo synthesis from basic precursors. In a separate study, jaesckeanadiol-3-p-hydroxyphenylpropanoate (**46**) (Supplementary Information Figure S3), a hemisynthetic ferutinin analogue of the naturally occurring sesquiterpene ester ferutinin, was shown to display higher potency against estrogen receptor-positive MCF-7 breast cancer cells as a result of induction of apoptosis and cell cycle arrest at the pre Go/G1 phase [49]. The IC_50_ value for the analogue was found to be 1μM, as compared to ferutinin, which demonstrated an IC_50_ value of 37 μM for MCF-7 breast cancer cells. Thus, hemisynthetic analogues provide novel alternatives for developing therapeutics to target cancer.

### 2.3. Synthetic Triazole Derivatives of Sesquiterpenes

Triazoles have been known for decades for their versatile pharmacological applications. There have been several reports on the derivatization of sesquiterpene lactones, including ludartin (**47**), parthenolide (**20**), and melampomagnolide B (**48**), which have been modified by the incorporation of the triazole moieties [34,50,51,52,53] to enhance their pharmacological activity, specifically antimicrobial, antitumor, and anti-inflammatory properties. In a recent study, isoalantolactone (**25**) derivatives containing the 1,2,3-triazole moiety were synthesized [54]. The authors indicated that the reactions of (**25**) and 4,15-epoxyisoalantolactone (**49**) SLs with hydrazoic acid proceeded with both regio- and stereo-selectively, which allowed for the synthesis of derivatives of (11*R*)-13-azido- or (11*R*)-11-amino-13-azido-11,13-dihydroisoalantolactones. Although the authors did not conduct any biological studies with these derivatives, these compounds open new avenues for developing targeted therapeutics for various types of cancers, given the structural transformation of the SLs.

More recently, a 1,2,3-triazole sesquiterpene derivative of β-himachalene (**50**), a bicyclic sesquiterpene [55], was synthesized by the dipolar cycloaddition of benzyl azide with α,β-unsaturated sesquiterpene ketones. The investigators found that the formed compounds displayed anticancer activities in vitro against fibrosarcoma (HT-1080), lung carcinoma (A-549), and breast cancer (MCF-7 and MDA-MB-231) cell lines. Furthermore, the compounds induced cell cycle arrest, particularly in the HT-1080 cell lines [56].

In a separate study, Poornima and co-workers isolated sesquiterpenes such as widdarol-peroxide (**51**), widdaranal C (**52**), and schisanspheninal A (**53**) along with the known guaianolide-type sesquiterpenes widdaranal A (**54**), widdaranal B (**55**), and isocuparenal **(56**) from *Schisandra grandiflora*. The chemical structures of sesquiterpenes (**47**) and (**48**) as well as (**51**), (**52**), (**55**), and (**56**) are shown in Supplementary Information Figure S4.

**Scheme 2:**
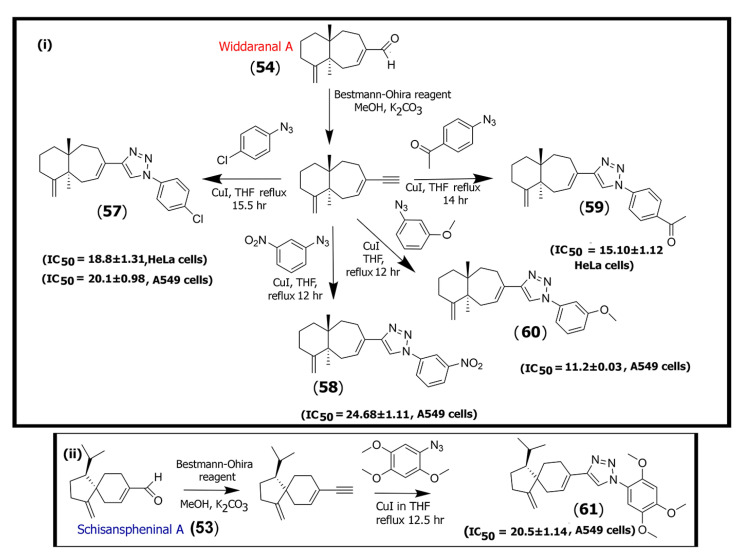
Schematic diagram showing the methodology for synthesizing triazole derivatives of (**i**) widdaranal A (**54**) and (**ii**) schisanspheninal A (**53**) that showed potency against HeLa and/or A549 tumor cells.

The group synthesized fourteen triazole derivatives from two of the isolated compounds (schisanspheninal A (**53**) and widdaranal A (**54**)) by first converting those to their respective acetylenes and then conjugating with various aromatic azide motifs under click chemistry conditions. The triazole derivatives were then tested against five cancer cell lines, including HeLa (cervical cancer), A549 (lung cancer), DU-145 (prostate cancer), MCF-7 (ER+ breast cancer), and B-16 (mouse melanoma) cell lines (Scheme 2). Their results showed that five of the synthesized azide derivatives (compounds (**57**) through (**61**) shown in Scheme 2) showed higher cytotoxicity toward HeLa and A549 cells, as demonstrated by IC_50_ values in the range of 15.1–24.6 μM [57], while relatively low cytotoxicity was observed for other cell lines. Thus, the triazole derivatives may have specificity toward those cells, and further testing will be necessary for exploring their development for therapeutics.

**Scheme 3:**
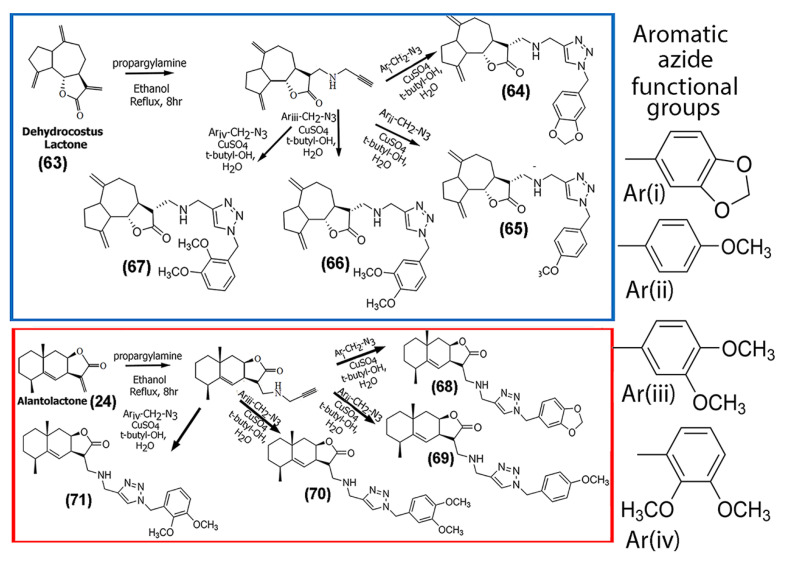
Schematic showing the methodology for synthesizing triazole derivatives of dehydrocostuslactone (**63**) (**top**) and alantolactone (**24**) (**bottom**) using click chemistry. (Adapted from [59]).

In a study conducted by Zhi and co-workers, the naturally occurring SL, 1-O-acetylbritannilactone (**62**), was modified with different azole groups, including triazole, oxadiazole, and imidazole, to create analogs (Supplementary Information Figure S5), and then the antifungal abilities of the compounds were probed [58]. While the antitumor ability was not tested, these newly synthesized derivatives may pave the way for further modifications with these functional groups for enhanced anticancer activity. In an interesting study, Artyushin and co-workers synthesized a series of derivatives of dehydrocostus lactone (**63**) and alantolactone (**24**), both of which are known for their anti-inflammatory and anticancer potential. The synthetic strategy employed cycloaddition reactions through click chemistry using two synthetic building blocks, azide and acetylene (Scheme 3) [59]. The authors reported that the triazole spacer not only functioned as a linker but also aided in interacting with the biological targets and increased the solubility of the SLs. The derivatives were tested for cytotoxicity against four cancer cell lines, including Hep-2 (squamous cell carcinoma of the larynx), HeLa, A549, and SH-SY5Y neuroblastoma cells. In general, most of the derivatives showed lower IC_50_ values for the SH-SY5Y and HeLa cell lines, while relatively higher IC_50_ values (> 50 μM) were observed for Hep-2 cells for most of the compounds tested. The IC_50_ values obtained for A549 cells were intermediate.

Overall, their results indicated that the synthesized dehydrocostus lactone triazole derivatives ((**64**), (**65**), (**66**), and (**67**) shown in Scheme 3) were relatively more potent against HeLa and SH-SY5Y cancer cell lines, while compounds (**64**) and (**67**) showed poor activity against Hep2 and A549 cells (IC_50_ values >100 μM). The corresponding triazole derivatives of alantolactone (**24**) (compounds (**68**), (**69**), (**70**), and (**71**) shown in Scheme 3) demonstrated weak cytotoxicity against the cancer cells tested in their study (Table 2), with IC_50_ values >70 μM in most cases. Given that dehydrocostus lactone in previous studies has been shown to suppress NF-kB activity as well as activate caspase 9, the authors predicted that the designed derivatives likely induced apoptosis in the cancer cells through similar pathways [60].

**Scheme 4:**
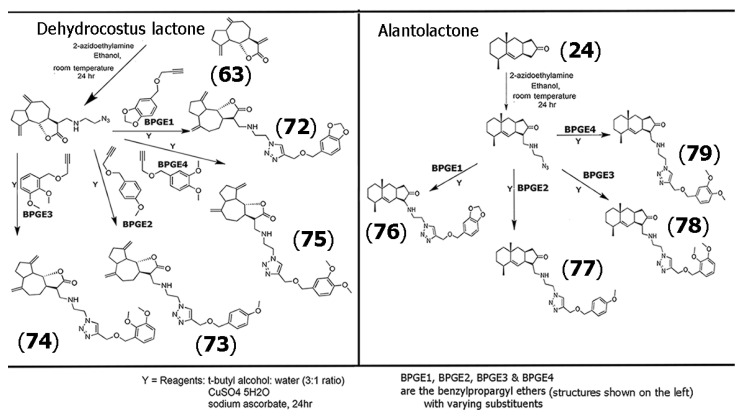
Methodology for the synthesis of triazole-alkoxybenzyl derivatives of dehydrocostus lactone (**63**) (**left**), yielding products (**72**) through (**75**), and alantolactone (**24**) (**right**), yielding products (**76**) through (**79**). Adapted from [63].

Alkoxybenzenes and arylidenes groups have also garnered tremendous importance in medicinal chemistry due to their applicability as highly viable pharmacophores [61,62]. Thus, several researchers have examined the use of these functional groups in developing scaffolds with SLs. As an expansion of the work described in Scheme 3, Neganova and co-workers synthesized a range of triazole derivatives of the SLs dehyrdocostuslactone (**63**) and alantolactone (**24**), where the azide group was attached to the SLs using azidoethylamine, and the resultant SL-azides were then treated with four different substituted benzyl propargyl ethers that were utilized to form the triazole-alkoxybenzyl derivatives of the SLs using “click chemistry” [63]. Scheme 4 illustrates the steps involved for the synthesis of the conjugates of dehydrocostus lactone (**63**), which yielded products (**72**) through (**75**). Compounds (**76**) through (**79**) were obtained for alantolactone derivatives synthesized using the same methodology.

**Table 2:** Summary of the bioactivity of selected synthesized derivatives of sesquiterpenes.

Synthesized SL-Derivatives	IC_50_ (μM)	Cell Line	In Vitro Assays	In Vivo Assays	Ref. #
(**50**)	10.03 33.2 27.2 36.7	HT-1080 A549 MCF-7 MDA-MB-231	MTT assay, apoptosis assay (flow cytometry) Cell cycle analysis	-	[55]
(**64**)	18.0 19.3 >100 >100	HeLa SH-SY5Y Hep-2 A549	MTT assay to test cytotoxicity		[59]
(**65**)	26.2 16.6 25.2 16.14	Hep-2 HeLa A549 SH-SY5Y	Cytotoxicity—MTT assay “ “ “	-	[59]
(**66**)	82.6 26.4 >100 21.83	Hep-2 HeLa A549 SH-SY5Y	Cytotoxicity—MTT assay “ “ “	-	[59]
(**67**)	>100 79.6 >100 89.5	Hep-2 HeLa A549 SH-SY5Y	Cytotoxicity—MTT assay “ “ “	-	[59]
(**68**) through (**71**)	>100 >72 >70 >56	Hep-2, HeLa, A549 SH-SY5Y	Cytotoxicity—MTT assay “ “ “	-	[59]
(**72**)	37.4 62.4 88.1 85.7	SH-SY5Y Hela Hep-2 A549	Cytotoxicity (MTT); mitochondria-dependent apoptosis and suppression of glycolysis process	Mitochondrial transmembrane potential *from rat liver was tested*	[63]
(**73**)	36.3 61.5 >100 >100	SH-SY5Y Hela Hep-2 A549	Cytotoxicity (MTT); mitochondria-dependent apoptosis and suppression of glycolysis process	Mitochondrial transmembrane potential from rat liver was tested	[63]
(**74**)	94.9 82.4 >100 >100	SH-SY5Y Hela Hep-2 A549	Cytotoxicity (MTT); mitochondria-dependent apoptosis and suppression of glycolysis process	Mitochondrial transmembrane potential from rat liver was tested	[63]
(**75**)	57.8 >100 >100 >100	SH-SY5Y Hela Hep-2 A549	Cytotoxicity (MTT); mitochondria-dependent apoptosis and suppression of glycolysis process	Mitochondrial transmembrane potential from rat liver was tested	[63]
(**76**)	33.6 38.9 55.7 29.3	SH-SY5Y HeLa Hep2 A549	Cytotoxicity (MTT); mitochondria-dependent apoptosis and suppression of glycolysis process	Mitochondrial transmembrane potential *from rat liver was disrupted*	[63]
(**77**)	41.7 55.1 67.1 47.3	SH-SY5Y HeLa Hep2 A549	Cytotoxicity (MTT); mitochondria-dependent apoptosis and suppression of glycolysis	Determination of mitochondrial transmembrane potential *from rat liver*	[63]
(**78**)	39.9 46.5 62.5 53.1	SH-SY5Y HeLa Hep2 A549	Cytotoxicity (MTT); mitochondria-dependent apoptosis and suppression of glycolysis	Determination of mitochondrial transmembrane potential *from rat liver*	[63]
(**79**)	45.2 81.0 91.0 83.5	SH-SY5Y HeLa Hep2 A549	Cytotoxicity (MTT); mitochondria-dependent apoptosis and suppression of glycolysis process	Determination of mitochondrial transmembrane potential *from rat liver*	[63]
(**80**)	8.6 10.01 9.97 16.41	MCF-7 SH-SY5Y HeLa IMR-32	MTT assay confirmed inhibition of cell proliferation. Glycolytic function in HeLa cells was assessed by a glycolysis stress test. Compounds (80) through (82) were identified as potential inhibitors of allosteric glycolytic enzyme and pyruvate kinase (PK) M2 oncoprotein. Docking studies revealed binding interactions. MTT assay confirmed inhibition of cell proliferation. Glycolytic function in HeLa cells was assessed by a glycolysis stress test. Some were identified as potential inhibitors of allosteric glycolytic enzyme and pyruvate kinase (PK) M2 oncoprotein.	-	[64]
(**81**)	8.17 7.93 22.68 5.76	MCF-7 SH-SY5Y HeLa IMR-32		-	
(**82**)	18.6 19.01 21.31 17.38	MCF-7 SH-SY5Y HeLa IMR-32		-	
(**83**)	8.91 22.75 21.06 8.64	MCF-7 SH-SY5Y HeLa IMR-32		-	
(**84**)	8.07 7.41 6.58 6.07	MCF-7 SH-SY5Y HeLa IMR-32		-	[64]

In general, the authors found that in the case of the alantolactones, when longer spacers were incorporated, cytotoxicity toward SH-SY5Y neuroblastoma tumor cell lines was enhanced, whereas the dehydrocostuslactone derivatives demonstrated an opposite effect, in that shorter spacers showed higher cytotoxicity, as was shown by the IC_50_ values of products (**72**) through (**79**), as shown in Table 2. The authors attributed the cytotoxicity effects observed to the fact that the conjugates not only impeded glycolysis but also successfully triggered a mitochondria-dependent apoptotic pathway in the tumor cells, which was confirmed by changes in the mitochondrial potential. In addition, the authors utilized “QikProp” to predict the ADME properties of the synthesized derivatives. It was predicted that in general, the percentage of oral absorption of the drug candidates was between 80 and 95% for most compounds, and LogP values were between 3.3 and 4.9, with a high degree of permeability through the blood–brain barrier indicating that the derivatives display drug-like properties.

More recently, the group created a new family of 3,5 bis(arylidene)-4-piperidone conjugates with SLs and demonstrated that the synthesized conjugates exhibited significantly higher cytotoxicity against tumor cells compared to the polyalkoxybenzyl derivatives [64]. With the SLs alantolactone (**24**), isoalantolactone (**25**), and dehydrocostuslactone (**63**), they used “click chemistry” to synthesize the conjugates. The chemical structures of some of the formed conjugates (compounds (**80**) through (**84**)) are shown in Supplementary Information Figures S6 and S7. Mechanistically, it was shown that these conjugates also altered the glycolytic pathway significantly and thereby inhibited cell proliferation of a variety of cancer cell lines, including SH-SY5Y, MCF-7, and IMR-32 (neuroblastoma), and HeLa tumor cells. They further conducted in silico studies, which revealed that the conjugates may act as potential inhibitors of the allosteric glycolytic enzyme pyruvate kinase MS oncoprotein. It is worth noting that prior to this work, 3,5 bis(arylidene)-4-piperidone was shown to act as a potent inhibitor of the viral protein dengue virus NS2B/NS3 protease [65]. Thus, the work by Neganova and colleagues demonstrates the importance of a target hopping approach, where similar motifs can be utilized for targeting multiple proteins and thereby act as dual targeting agents.

**Scheme 5:**
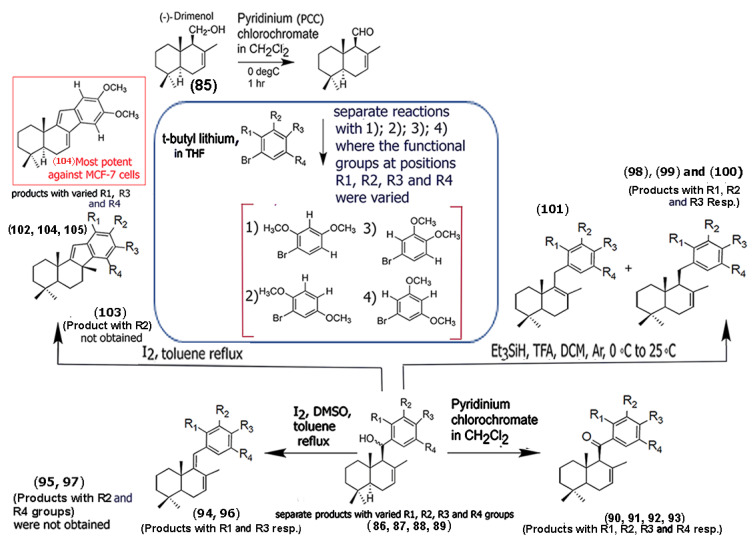
Structures of chemical derivative building blocks strategically developed by functionalizing with a variety of dimethoxyaryl groups using the sesquiterpene alcohol with 7-(-) drimenol (**85**) as the starting point. The most potent compound against MCF-7 breast cancer cells is shown in the red box. (Adapted from [66]).

In a recent study, Araque and co-workers synthesized dimethoxyaryl–sesquiterpene derivatives that demonstrated cytotoxicity against MCF-7 breast cancer cells [66]. Specifically, the group utilized the sesquiterpenoid alcohol drimenol (**85**), obtained from hexane extracts of the bark of *Drimys winteri*, and carried out a series of modifications to the sesquiterpene and introduced a variety of aryl scaffolds by replacing the -CH_2_ group on C-11 with the aryl moieties through linkers (Scheme 5). The authors ensured that the double bond in the drimane skeleton remained unchanged for all of the derivatives. Furthermore, the authors found that incorporating dimethoxy groups in the aryl segments played a critical role in enhancing cytotoxicity toward MCF-7 breast cancer cells. Among the various derivatives synthesized (compounds (**86**) through (**105**)), compound (**104**) was the most potent among all the derivatives, showing a higher selectivity toward cancer cells compared to the known chemotherapeutic drug daunorubicin. Compounds (**95**), (**97**), and (**103**) did not form, indicating that not only the aryl methoxy groups utilized, but also the reagents (in this case, the presence of solvent DMSO) used for modification can alter product composition and yields. Mechanistically, the authors showed that the compound successfully increased ROS levels in tumor cells and selectively inhibited the activity of topoisomerase II, thereby causing apoptosis of MCF-7 breast tumor cells. These results are promising and lay the groundwork for developing novel sesquiterpene aryl derivatives for enhanced antitumor activity.

Several studies have also indicated that sesquiterpene lactones are capable of inhibiting cyclooxygenase-2 (COX-2) activity [67]. In an interesting study conducted by Coricello and co-workers, the SLs α-santonin (**106**) and α-desmotroposantonin (**107**) (Supplementary Information Figure S8) and their derivatives were investigated for the treatment of inflammation via the inhibition of COX-2 activity and expression. Since inflammation plays a key role in triggering and manifesting several diseases, including cancer [68], it is important to investigate the anti-inflammatory effects of SLs and their derivatives. The authors proposed the creation of O-benzyl ether derivatives and oxo-phenyl-ethyl derivatives of α-desmotroposantonin and examined the effects of different substituents. They carried out molecular docking studies with the COX-2 enzyme and determined that the phenyl ring of the benzyl ether derivative played a key role in hydrophobic and stacking interactions with the VAL523 and ALA516 residues within the binding pocket of the enzyme. They synthesized the optimal compounds and found that two of the oxo-phenyl-ethyl derivatives demonstrated stronger reduction in the expression of COX-2 at a lower concentration compared to the parent SLs. Furthermore, the compounds also showed promising results against prostaglandin E2, which is also known to play a key role in inflammation. We believe that it is worth conducting further studies using these derivatives for possible tumor cell targeting, as COX-2 activity is upregulated in many cancers [69].

**Figure 3:**
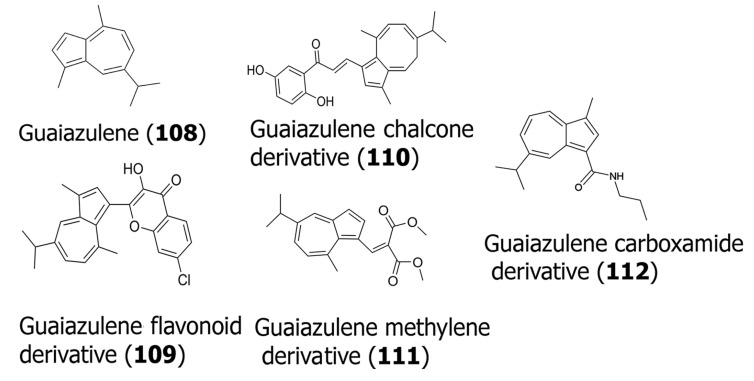
Structures of guaiazulene (**108**) and its synthesized derivatives that have shown potent activity (**109**–**112**).

The sesquiterpene guaiazulene (**108**) [70] has also been modified by many researchers to create potent anticancer compounds. For example, Ma and co-workers recently developed [71] a series of conjugates by incorporating cytotoxic methylene groups, chalcone, sulfonamide, or flavonoid groups. The most potent compounds developed that were found to be cytotoxic to K562 (chronic myelogenous leukemia) and MDA-MB-231 cells are shown in Table 3, and their structures are shown in Figure 3. In general, the flavonoid derivative (7-chloro-3-hydroxy-2-(5-isopropyl-3,8-dimethylazulen-1-yl)-4H-chromen-4-one) (**109**) was found to show cytotoxicity toward both cell lines. The chalcone derivative (**110**) ((E)-1-(2,5-dihydroxyphenyl)-3-((3aZ,6E,8Z)-6-isopropyl-3,9-dimethyl-5H-cyclopenta [8]annulen-1-yl)prop-2-en-1-one) and the methylene derivative (**111**) (dimethyl 2-((5-isopropyl-8-methylazulen-1-yl)methylene) malonate) showed significantly higher cytotoxicity toward the K562 cell line, implying specificity. The compounds were also found to demonstrate potent anti-inflammatory and antiviral properties. Several guaiazulene–carboxamide derivatives have also been synthesized, where 3-methyl, 7-isopropyl-3-methyl, or 2-methoxy groups were conjugated with the guaiazulene moiety [72]. In particular, the guaiazulene–carboxamide derivative (7-isopropyl-3-methyl-N-propylazulene-1-carboxamide) (**112**) showed relatively higher cytotoxicity with oral squamous cell carcinoma (OSCC) cells. Overall, the carboxamide derivatives revealed higher IC_50_ values with OSCC cells for the lead compound that was synthesized (**112**) compared to the methylene and the flavonoid or chalcone functionalized guaiazulene derivatives with K562 and MDA-MB-231 cells. These studies, however, are promising, as the lead compounds obtained can be further modified to develop novel, more potent derivatives of guaiazulene for therapeutic applications.

### 2.4. Mercapto Derivatives of Sesquiterpenes and Their Antitumor Activity

Zerumbone (**113**), a sesquiterpene obtained from the rhizome of *Zingiber zerumbet,* has an eleven-member ring system that contains a pentadienone moiety that has been shown to be essential for its antitumor activity [73].

**Figure 4:**
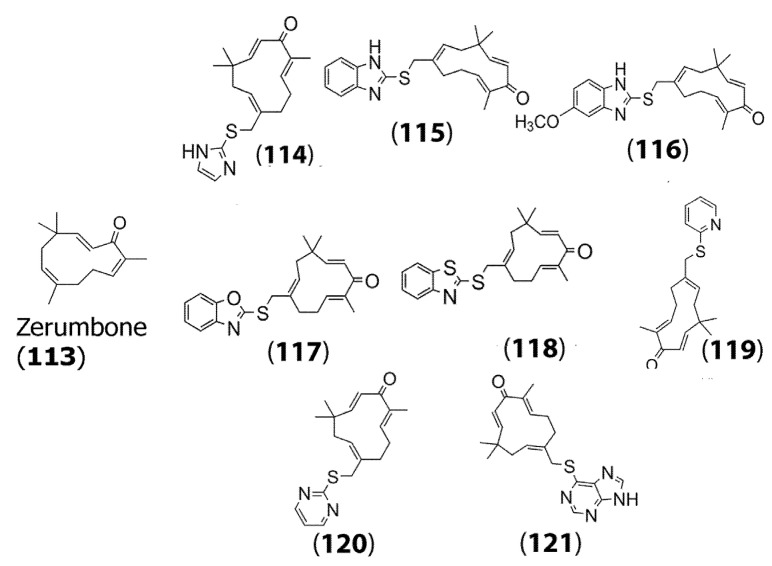
Chemical structures of zerumbone (**113**) and some of its mercapto derivatives synthesized: mercaptoimidazole derivative (**114**); mercaptobenzimidazole derivative (**115**); mercaptomethoxy benzimidazole derivative (**116**); mercaptobenzoxazole derivative (**117**); mercaptobenothiazole derivative (**118**); mercaptopyridine derivative (**119**); mercaptopyrimidine derivative (**120**); mercaptopurine derivative (**121**). Adapted from [75].

While it displays anticancer activity through several mechanisms in a wide array of tumor cell types, efforts have been made to enhance its antitumor activity by further functionalization [74]. In a recent study conducted by Hieu and co-workers, a variety of mercapto zerumbone derivatives were prepared by adding C-S-C linkers at the C15 position of the sesquiterpene. Specifically, the group designed and synthesized several compounds by incorporating functional groups, including mercaptoimidazole (**114**), mercaptobenzimidazole (**115**), methoxy-2-mercaptobenzimidazole (**116**), mercaptobenzoxazole (**117**), mercaptobenzothiazole (**118**), mercaptopyridine (**119**), mercaptopyrimidine (**120**), and mercaptopurine (**121**) to form zerumbone derivatives (Figure 4). The authors then tested the cytotoxicity of the designed compounds on three cell lines, namely, HepG2 cells, HeLa cervical cancer cells, and A549 lung cancer cells. Their results indicated that many of the derivatives showed higher cytotoxicity toward the cancer cells compared to the parent zerumbone (**113**), with remarkably lower IC_50_ values, which is promising [75]. For example, (**116**) showed an IC_50_ value of 0.93 μM for A549 cells compared to zerumbone, which showed an IC_50_ value of 6.78 μM.

**Scheme 6:**
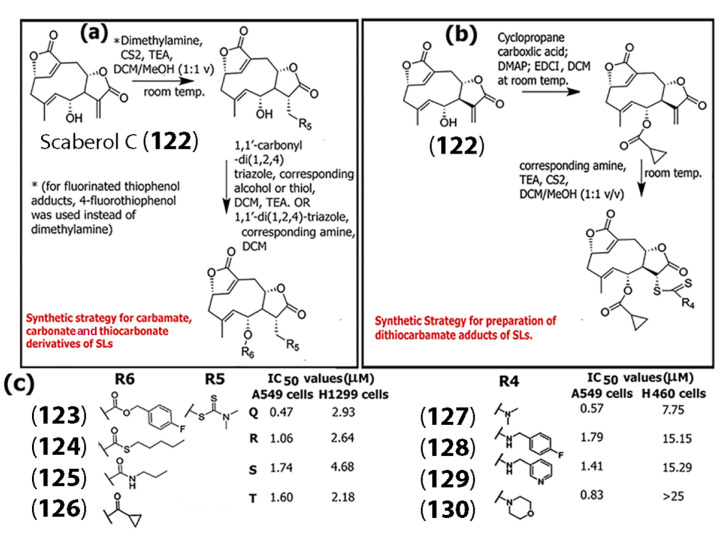
(**a**) Synthesis of carbamate, carbonate, or thiocarbonate derivatives of sesquiterpene lactones using the SL scaberol C (**122**) as an example. (**b**) Synthesis of dithiocarbamate adducts. (**c**) The chemical structures of substituents R5 and R6 that resulted in the most potent compounds against A549 and H1299 cell lines (**left**); and chemical structures of substituent R4 that resulted in the most potent compounds against the proliferation of A549 and H460 cell lines (**right**) (Adapted from [76]).

In another study, Song and co-workers designed and synthesized ROS-responsive monomeric and dimeric thiol adducts of scaberol C (**122**), a germacrane SL, [76] and examined the activity of those pro-drugs for TrxR1 (thioredoxin reductase 1) inhibitory activity and their impact against non-small cell lung cancer (NSCLC) cell lines, including H1299, A549, and H460 (Scheme 6). In general, TrxR1 is responsible for maintaining redox homeostasis by regulating the Nrf2-Keap1 response system in cells [77]. Based on initial results, further modifications were carried out by introducing different carbamate, carbonate, or thiocarbonate side chains (Scheme 6a,b).

The group also synthesized 4-fluorothiophenol adducts. Overall, their results indicated that most of the derivatives synthesized showed higher potency against A549 cells. (Scheme 6c) shows the structures of the substituents utilized that resulted in the most potent anticancer compounds (**123**), (**124**), (**125**), and (**126**), as demonstrated by the IC_50_ values obtained. In general, the IC_50_ values of several of the compounds synthesized by the authors showed lower IC_50_ values (in the range of 0.47–2.80 μM for A549 cells) compared to the known chemotherapeutic oxaliplatin (IC_50_ = 7.42 for A549 cells). For H1299 cells, the IC_50_ values were found to be in the range of 2.18–5.37 μM for the compounds, while treatment with oxaliplatin resulted in IC_50_ values of 8.49 μM, which is very promising. Compounds (**127**), (**128**), (**129**), and (**130**) were also highly potent against A549 cells but showed relatively less potency against H460 cell lines.

In general, the researchers in this study considered the fact that tumor cells contain higher levels of ROS (reactive oxygen species), and thioether bonds containing prodrugs tend to become oxidized by ROS and hydrolyze easily to release the active compounds [78] that can inhibit the growth of the tumor cells. Their results were encouraging. They found that of the series of prodrugs studied, one of the prodrugs (**125**) containing an alkyl amide group showed the most potent anti-NSCLC activity and efficiently released the active compound from the prodrug, which then formed a covalent bond with a novel Cys475 site and a known Sec498 site of TrxR1. This allowed for the modulation of TrxR1 activity and lowered its expression, ultimately resulting in a burst of ROS that led to NSCLC cell apoptosis and ferroptosis. More importantly, this compound also demonstrated toxicity against NSCLC organoids and xenograft tumor models in mice.

Interestingly, scaberol C (**122**) was also recently utilized for the preparation of amino acid ester-trifluoroacetate derivatives that demonstrated inhibitory activity against A549 and H460 NSLC cells. One of the compounds (compound (**131**)) (Supplementary Information Figure S11) synthesized showed an IC_50_ value of 6.21 μM, which was lower than the known chemotherapeutic drug cisplatin [79].

**Figure 5:**
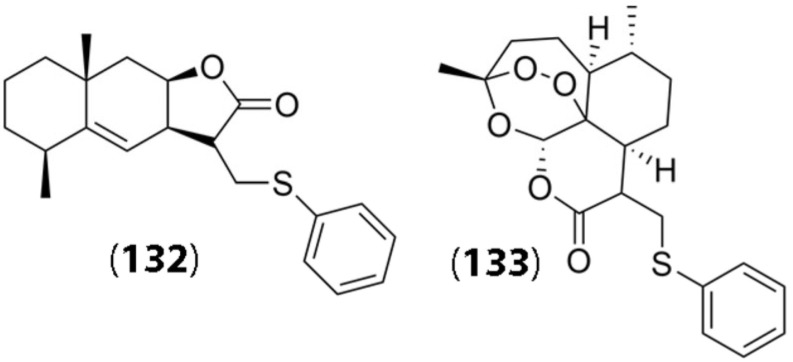
Chemical structures of synthesized thiophenol derivatives of sesquiterpene lactones. Compound (**132**) indicates the structure of alantolactone derivative, and compound (**133**) displays the structure of the corresponding Artemisinin derivative. (Reproduced with permission from Springer, [80]).

In another study, several sesquiterpene lactones isolated from plant sources, including *Sausserea lappa*, *Tanacetum patenium* leaves, *Centaurea macrocephala*, and *Inula helenium* L., were utilized for chemical modification and preparation of thiophenol derivatives. Some of the SLs used were obtained from parent lactones semi-synthetically and were conjugated with thiophenol groups in an effort to design prodrugs that were capable of tumor cell targeting [80]. The thiophenol groups were conjugated through Michael addition in the presence of triethylamine. Interestingly, the thiophenol conjugates were found to demonstrate lower cytotoxicity compared to the SLs themselves; however, they could act as prodrugs for the specific lactones. It was observed that the sulfide group of the thiophenol SL conjugates was readily oxidized by ROS, which led to the formation of sulfinic or sulfonic acid and the SL. Once again, an important aspect of these reactions involved the presence of elevated levels of ROS in tumor cells that allowed for the reaction to occur, thereby leading to higher cytotoxicity in tumor cells. The researchers found that, in general, several of the prodrug conjugates showed moderate cytotoxicity against the tumor cells. Of the several conjugates synthesized, thiophenol conjugates of alantolactone (**24**) and artemisinin (**22**) specifically showed higher cytotoxicity against cancer cell lines, including embryonal rhabdomyosarcoma (RD), HeLa, and HCT 116 colon cancer cells. Their chemical structures (compounds (**132**) and (**133**)) are shown in Figure 5. The IC_50_ values for RD cells, HeLa, and HCT116 cells for compounds (**132**)/(**133**) were found to be 18.4 μM/14.9 μM, 34.3 μM/5.82 μM, and 31.7 μM/33.9 μM, respectively. Thus, compound (**133**) showed significantly higher efficacy toward HeLa cells compared to compound (**132**). Further functionalization of these thiol adducts may enhance selectivity and potency toward tumor cells.

### 2.5. Conjugates of Sesquiterpene Lactones and Their Chemosensitization Ability

Recently, Meng and co-workers reported the isolation and discovery of amino acid conjugates of sesquiterpene lactones from chicory roots of the plant species *Cichorium intybus* L. Specifically, compounds such as 11β, 13-dihydro-13-prolyl-lactucin were found to demonstrate strong anti-inflammatory activity, as was evidenced by the suppression of expression of pro-inflammatory growth factors, such as TNF-α, COX-2, and interleukin-6 (IL-6) [81]. While no cancer cell studies were conducted with those amino acid conjugates, that would be an interesting next step. Furthermore, additional derivatives of the compounds may also be developed.

**Figure 6:**
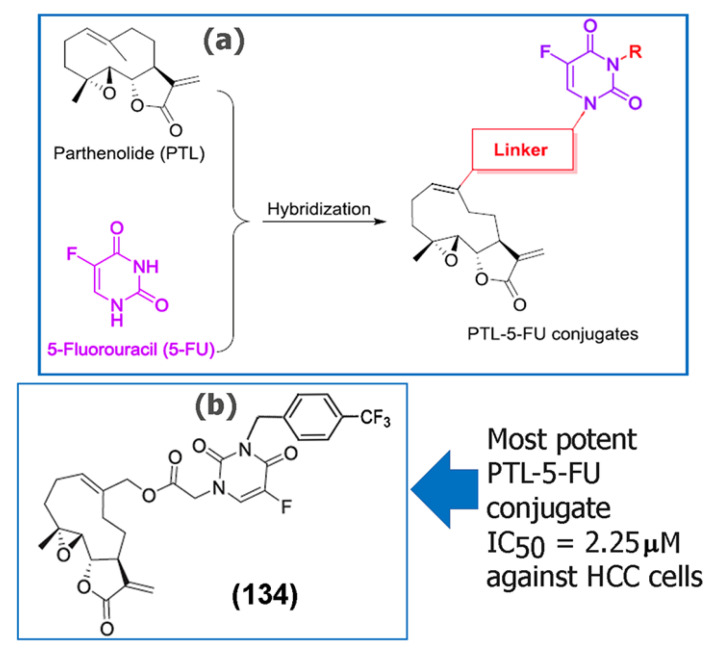
(**a**) Design strategy developed for the synthesis of a 5-fluorouracil conjugate of PTL (**20**). (**b**) Chemical structure of the most potent conjugate (**134**) synthesized. Reproduced with permission from Elsevier, [86].

Although SLs have shown potent anticancer activity against a variety of tumor cell lines, the overall bioavailability of SLs has been relatively poor, in some cases limiting their clinical applications [82]. Additionally, some SLs have also been reported to be toxic depending upon the structural aspects, mode of consumption, and the components of the extract itself. Some of the mechanisms of toxicity have been reviewed thoroughly by Amorim and co-workers [83]. Thus, researchers have developed several analogues of a variety of SLs [84,85] as well as chemotherapeutic drug conjugates to improve therapeutic effects. For example, Ding and co-workers recently synthesized a series of 5-fluorouracil (5-FU) conjugates of the SL PTL (**134**), (**135**), (**136**), (**137**), and (**138**) (Table 4). Of those, several of the conjugates demonstrated higher cytotoxicity against the fluorouracil-resistant human hepatocellular carcinoma (HCC) cell line (Bel-7402/5-FU) compared to the precursor SL PTL (**20**) [86]. The researchers concluded that the substituent on the 3-N-5-FU moiety of PTL-5-FU conjugates was important for targeting tumor cells. Additionally, the benzyl group at the 3-N-5-FU moiety was shown to successfully incorporate a variety of substituents, and in general, the substituted benzyl groups were more favored than aliphatic groups. Furthermore, the most potent conjugate (**134**) (Figure 6) induced apoptosis in the 5-FU HCC tumor cells and successfully reversed drug resistance by inhibiting pathways such as MDR1, ABCC1, and ABCG2 that lead to the expulsion of drugs from the intracellular environment.

In another study, the chemotherapeutic drugs doxorubicin and daunorubicin were conjugated with several SLs through N-alkylation. The SLs studied included alantolactone (**24**), alloalantolactone (**139**), epoxy-isoalantolactone (**140**), epoxyalloalantolactone (**141**), 6-hydroxyxanthanodiene (**142**), and alanthodiene (**143**) (Supplementary Information Figure S10). The group tested the cytotoxicity of the conjugates with a variety of cancer cell lines, including A549, HCT116 (colon cancer cells), MCF-7, and RD (rhabdomyosarcoma) cells. Their results showed comparable cytotoxicity toward the tumor cells to that of the chemotherapeutics alone, and there was no increase in cytotoxicity toward non-cancer cells. Additionally, a few of the conjugates enhanced the viability of normal human renal epithelial cells [87]. In vivo studies on rats revealed that the cardiotoxicity of several of the conjugates was lower compared to the neat drugs; thus, conjugating chemotherapeutics with sesquiterpenes may help potentiate lower cytotoxicity toward non-cancer cells. The bioactivities of some of the selected drug conjugate compounds (**144**) and (**145**) with sesquiterpenes alantolactone (**24**) and isoalantolactone (**25**) [88] are also provided in Table 4. The chemical structures of selected chemotherapeutic drug conjugates with PTL and ATL are shown in Supplementary Information Figure S11.

In addition to the enhanced activity of sesquiterpene drug conjugates, SLs such as xanthatin (**146**) and costunolide (**34**) have been shown to sensitize chemotherapeutic drugs, thereby enhancing their efficacy against tumor cells [89]. For example, Chen and co-workers demonstrated that when the SL costunolide (**34**) was administered with doxorubicin, results showed higher apoptosis in prostate cancer cells by not only increasing ROS in the tumor cells but also by affecting mitogen-activated protein kinase (MAPK) pathways more efficiently compared to doxorubicin alone [90].

In an exciting study conducted by Di Giacomo and co-workers, caryophyllane sesquiterpenes such as β-caryophyllene (BC) (**8**) and β-caryophyllene oxide (BCO) (**147**) have also been found to sensitize the effects of the kinase inhibitor drug sorafenib against hepatocellular carcinoma (HCC), cholangiocarcinoma (CCA), and pancreatic ductal adenocarcinoma (PDAC) cell models. Results were particularly promising against pancreatic Bx-PC3 adenocarcinoma cells with BCO [91]. The authors indicated that a combination of BCO or BC with sorafenib resulted in a three to four-fold downregulation of the multidrug-resistant proteins MRP1 and MRP2. Additionally, substantial downregulation of the phosphorylation of (Tyr705)-STAT3 compared to sorafenib alone was observed. Their results further reinforce the chemo-sensitizing effects of sesquiterpenes and indicate that sesquiterpenes may be particularly applicable in the context of drug resistance often observed upon administration of chemotherapeutics. The chemical structures of (**146**) and (**147**) are shown in Supplementary Information Figure S12.

### 2.6. Anticancer Activity of Sesquiterpene Quinones and Their Derivatives

**Figure 7:**
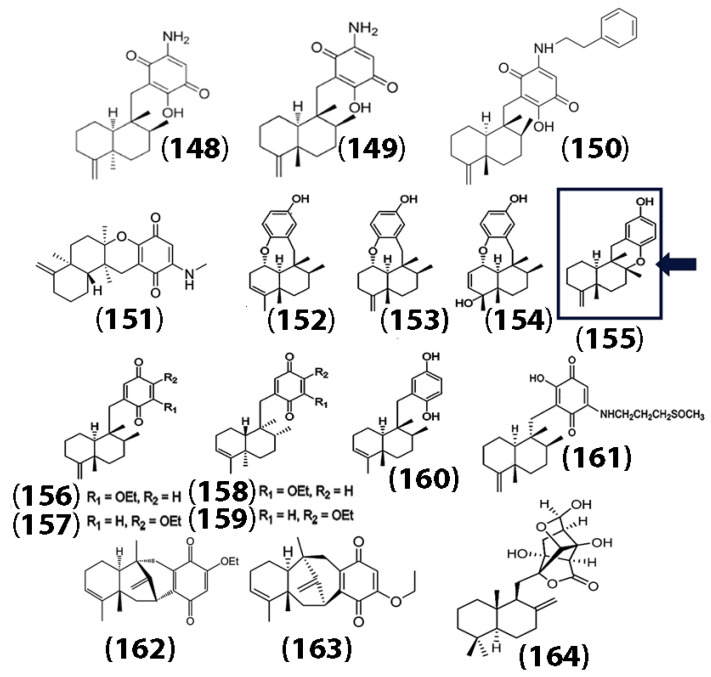
Chemical structures of marine sponge-derived sesquiterpene quinols and sesquiterpene quinones. Dysquinol D (**155**) is shown in the box. The ether bridge between C8 and C17 is shown with an arrow. Adapted and reproduced with permission from the Royal Society of Chemistry, [94].

In addition to sesquiterpene lactones, sesquiterpene quinones (SQ) have garnered importance due to their exceptional bioactivity [92]. Naturally derived sesquiterpene quinones and hydroquinones from marine sources, including 5-epi-smenospongine (**148**), smenospongine (**149**), and smenospongiadine (**150**), have been shown to have potency against multiple cancer cell lines, including U937 monocytic leukemia, HeLa cervical cancer, and HepG2 liver cancer cell lines, with higher cytotoxicity toward the U937 cells [93]. In addition, one of the isolated compounds, 20-demethoxy-20-methylaminodactyloquinone (**151**) (Figure 7), also showed a high affinity toward the cyclin-dependent kinase CDK-2 enzyme, which plays a crucial role in the regulation of cell cycles. In a separate study, Jiao and co-workers isolated sesquiterpene dysquinols and quinones from the marine sponge *Dysidea avara*. The structures of the isolated compounds ((**148**) through (1**59**)), the anticancer compound avarol (**160**), and additional sesquiterpene quinones derived from marine sponges (langcoquinone (**161**), dysidavarone A (**162**), dysidavarone D (**163**),herqupenoid A (**164**)) are also shown in Figure 7 [94,95,96,97].

Interestingly, all of the quinones showed cytotoxicity against human melanoma cancer cells (NCI-H929 cell lines), while only one of the quinols (**155**) showed both cytotoxicity against NCI-H929 cells and strong inhibitory activity against NF-κB. The authors indicated that the ether bridge (indicated by the arrow) of dysquinol D (**155**) may play a crucial role in the cytotoxicity toward the cancer cells. Selected sesquiterpene quinones and their bioactivities are summarized in Table 5.

Another potent sesquiterpene, seriniquinone (**165**), which exhibits an entirely fused aromatic ring structure, was initially isolated from marine bacteria and was shown to demonstrate potent antitumor activity against melanoma [98]. However, the compound itself was largely insoluble, and therefore several analogues have been developed based on structure–activity studies to enhance its efficacy, and further studies are underway. To further enhance the activity of sesquiterpene quinones, SQs have also been conjugated with proteins. In a pioneering study, Novaković and co-workers created modified conjugates of the enzyme lysozyme with the sesquiterpene quinone avarone (**166**) and its derivatives [99]. The conjugates were shown not only to retain the antibacterial activity of lysozyme but also demonstrated synergistic activity where the enzyme not only targeted the bacterial cell walls but also was able to deliver the quinone into the bacteria, which can cause DNA damage in bacteria, thereby enhancing their antibacterial activity. Furthermore, some of the conjugates were found to be active against both Gram-positive and Gram-negative bacteria. While no antitumor cell studies were conducted, that would be an interesting next step for these derivatives. The chemical structures of compounds (**165**) and (**166**) are shown in Supplementary Information Figure S13.

## 3. Nanoformulations of Sesquiterpenes

Due to the high surface to volume ratio of nanoscale materials, several researchers have explored biomimetic synthesis of nanoparticles, as well as the utility of nanoparticles fused with natural products such as polyphenols and terpenes for biomedical applications [100,101]. In a recent study, Rajendran and co-workers created mono- and di-terpene fused silver nanoparticles (AgNPs) and demonstrated that the fused particles successfully inhibited the viability of *N. fowleri* cysts and reduced the cytopathogenicity of *N. fowleri* trophozites to keratinocytes (HaCaT cells). The authors concluded that such natural product fused NPs may alleviate *N. fowleri*-related amoebic infections [102]. The SL alantolactone (**24**) has been shown to enhance the apoptotic activity of the chemotherapeutic drug cisplatin. In a study conducted by Alipour and colleagues, the researchers found that when a nanoformulation of zinc oxide nanoparticles was used in conjunction with the chemotherapeutic drug cisplatin and alantolactone (**24**), the drug response ability of cisplatin was significantly enhanced in ovarian cancer cells. Not only were ROS levels in SKVO3 ovarian cancer cells increased, but the expression of XIAP (X-linked inhibitor of apoptosis), cyclin D1, and BCL2 genes was reduced upon treatment with the triple combination, leading to increased cytotoxicity of SKOV-3 cells [103]. These results are promising, especially given that new drug formulations toward the treatment of cancer are essential for increasing treatment efficacy and targeting ability.

In a fascinating study carried out by Ran and co-workers, the group created a “Y-shaped” all-D peptide (DWVAP) that was successful in all-stage precision glioma targeting. The group developed polyethylene glycol-poly lactic acid (PEG-PLA) micellar nanoparticles that were then decorated with the peptide sequence that was capable of crossing the blood–brain barrier in mice models [104]. The micelles were loaded with either the SL PTL (**20**) or the chemotherapeutic drugs paclitaxel and temozolomide. When treated with a combination of nanomicelles loaded with PTL (**20**), the group demonstrated that parthenolide significantly enhanced the efficacy of paclitaxel through NF-κB inhibition. Thus, this novel drug carrier not only targeted glioma cells efficiently but also showed efficient drug delivery and demonstrated blood–brain barrier-penetrating ability. Interestingly, PTL (**20**) has also been loaded into poly(styrene-alt-maleic anhydride)-b-poly(butyl acrylate) (PSMA-b-PBA) amphiphilic diblock copolymer-based micelles and demonstrated sustained release of PTL. However, compared to the chemotherapeutic doxorubicin, the micelles released PTL more rapidly. The interactions between PTL and the micelles were largely attributed to hydrophobic interactions [105].

**Figure 8:**
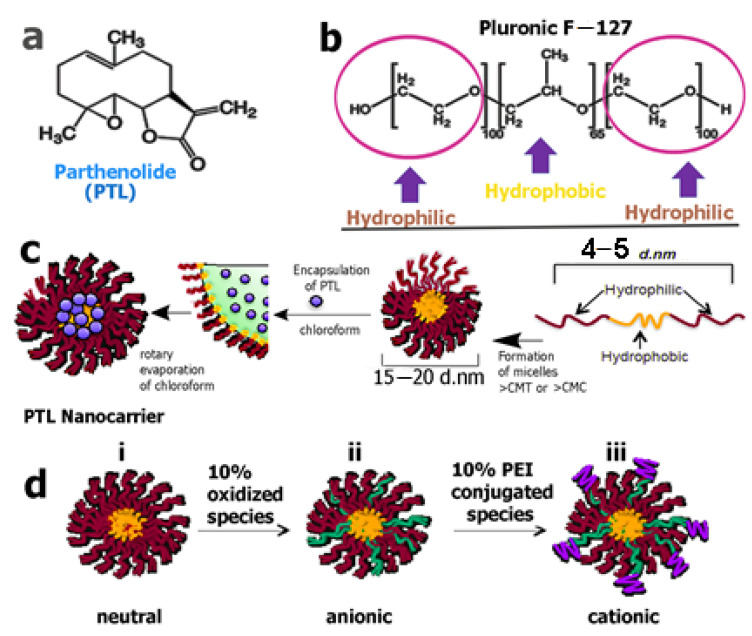
(**a**) The chemical structure of the SL parthenolide. (**b**) Triblock co-polymer structure of pluronic F−127. (**c**) Scheme showing the preparation of parthenolide-loaded micelle formation. (**d**) Micelle systems: (i) neutral; (ii) anionic (oxidized); (iii) cationic (PEI-conjugated). PEI = polyethylenimine. (Reproduced with permission from *American Chemical Society* [106]).

Deller and co-workers designed a variety of pluronic acid-based micellar structures as vectors for solvent-free delivery of PTL (**20**) for the treatment of acute lymphoblastic leukemia (ALL) [106]. They created micelles (Figure 8) of varied charges and found that the F127 pluronic–PTL-loaded micelles were the most effective in the treatment of patient samples, demonstrating a higher level of cytotoxicity toward bone marrow-derived diagnostic primary T-ALL patient cells. Their results suggested that PTL-loaded F127 micelles could be used instead of a co-solvent PTL for delivery to primary T-ALL patient cells.

In another study, the steroidal molecule lithocholic acid was self-assembled into nanotubes and encapsulated with guaiane-type sesquiterpenes such as aguerin B (**167**), cynaropicrin (**168**), and grosheimin (**169**) (Supplementary Information Figure S14) in order to enhance their delivery [107]. The researchers found that encapsulation of the sesquiterpenes in the nanotubes yielded better results, showing phytotoxicity against weed growth. Although no mammalian cells were tested, those experiments may yield interesting results as well and are worth an investigation.

Thus, sesquiterpenes and their numerous derivatives provide a vast resource of natural products that may be developed for the treatment of cancer. Further development, particularly functionalization and nanoscale formulations for targeted therapeutics, may accentuate the use of these compounds.

## 4. Clinical Trials

To further elucidate the therapeutic potential of sesquiterpenes and their derivatives, several of the naturally derived and chemically modified derivatives have undergone clinical trials (http://www.clinicaltrials.gov, accessed on 3 July, 2025) over the past seventy years, though most of the clinical trials have focused on cancer or antiviral studies with naturally derived sesquiterpenes and SLs such as thapsigargin (**21**), dehydrocostuslactone (**63**), arglabin (**170**), parthenolide (**20**), costunulide (**34**), alantolactone (**24**), and atracytylenolide (**171**), to name a few [108]. In a randomized, double-blind, placebo-controlled study for oral artesunate therapy in patients with colorectal cancer, the artemisinin derivative artesunate (**172**) showed that more than 60% of the patients in the artesunate group demonstrated a significant decrease in the expression of cell proliferation biomarkers, such as Ki67. Furthermore, the probability of recurrence-free survival was higher among artesunate-treated patients compared to a placebo. The authors determined that patients with abstruse distribution of colorectal cancer can potentially have better outcomes from systemic neo-adjuvant therapy, and that artesunate may aid in treatment, as it does not delay surgery [109]. This is promising, given that artesunate has also shown potency against a variety of tumor cells, including ovarian cancer (IC_50_ values of 26.9 μM for UWB1 cells, 15.2 μM for Caov-3, and as low as 4.7 μM for OVCAR-3 cells) [110] and non-small cell lung cancer cell lines, such as H1975 (IC_50_ = 4.02 μM) and H460 cells (16.11 μM), at 48 h [111] in addition to other tumor cell types. In another study, phase II clinical trials with the semi-synthetic sesquiterpene irofulven (**173**) (IC_50_ of 50 nM against epithelial ovarian MA148 tumor cells) [112] in patients with recurrent gynecologic cancers who had previously undergone platinum-based chemotherapy were carried out, where patients were administered the drug daily for 28 days. However, while the drug showed the desired activity, it also manifested several toxicities that induced side effects, such as nausea with emesis and electrolyte disbalance in addition to thrombocytopenia, neutropenia, and renal tubular acidosis. Thus, the authors concluded that while irofulven (**173**) cannot be administered as a single treatment, it may be administered with other agents, such as angiogenesis inhibitors or with PARP inhibitors or Bcl-2 antagonists [113]. Nevertheless, irofulven (**173**), which is a chemical analog of the fungi-derived sesquiterpene illudin S (**174**) (IC_50_ of 14 nM against SW-480 colorectal cancer cells) [114], was relatively less toxic compared to illudin S (**174**), for which clinical trials had to be halted due to toxicity issues. In a promising study, mipsagargin (G-202) (8-O-(12-aminododecanoyl) 8-O-debutanoyl thapsigargin)-D-γ-E-γ-E-γ-EEOH) (**175**), a thapsigargin-derived prodrug acted as a SERCA pump protein inhibitor, with high specificity toward PSMA-overexpressing prostate cancer cells. It has undergone phase I clinical trials in patients with refractory or advanced solid metastatic tumors. Results showed that while some patients developed adverse side effects, such as fatigue, nausea, rash, and elevated creatine levels, a few patients had renal failure that was reversible. While no clinical response was observed, overall, the authors concluded that the drug was generally tolerated by the patients and showed disease stabilization in a subset of patients [115]. More recently, phase II clinical trials as a second line of therapy in patients with advanced hepatocellular carcinoma (HCC) that had progressed after treatment with the drug sorafenib were conducted. Results demonstrated that the drug was well tolerated and promoted long-term disease stabilization in patients with advanced HCC [116]. In general, relatively fewer studies on chemically modified derivatives are currently available, and further clinical trials to examine the efficacy of many of the drug molecules discussed in this review will be necessary to determine their therapeutic efficacy. The chemical structures of compounds (**170**) to (**175**) are shown in Supplementary Information Figure S15.

## 5. Sesquiterpenes as Potential Treatment for Neurodegenerative Diseases

### 5.1. Pterosin Sesquiterpenes

**Figure 9:**
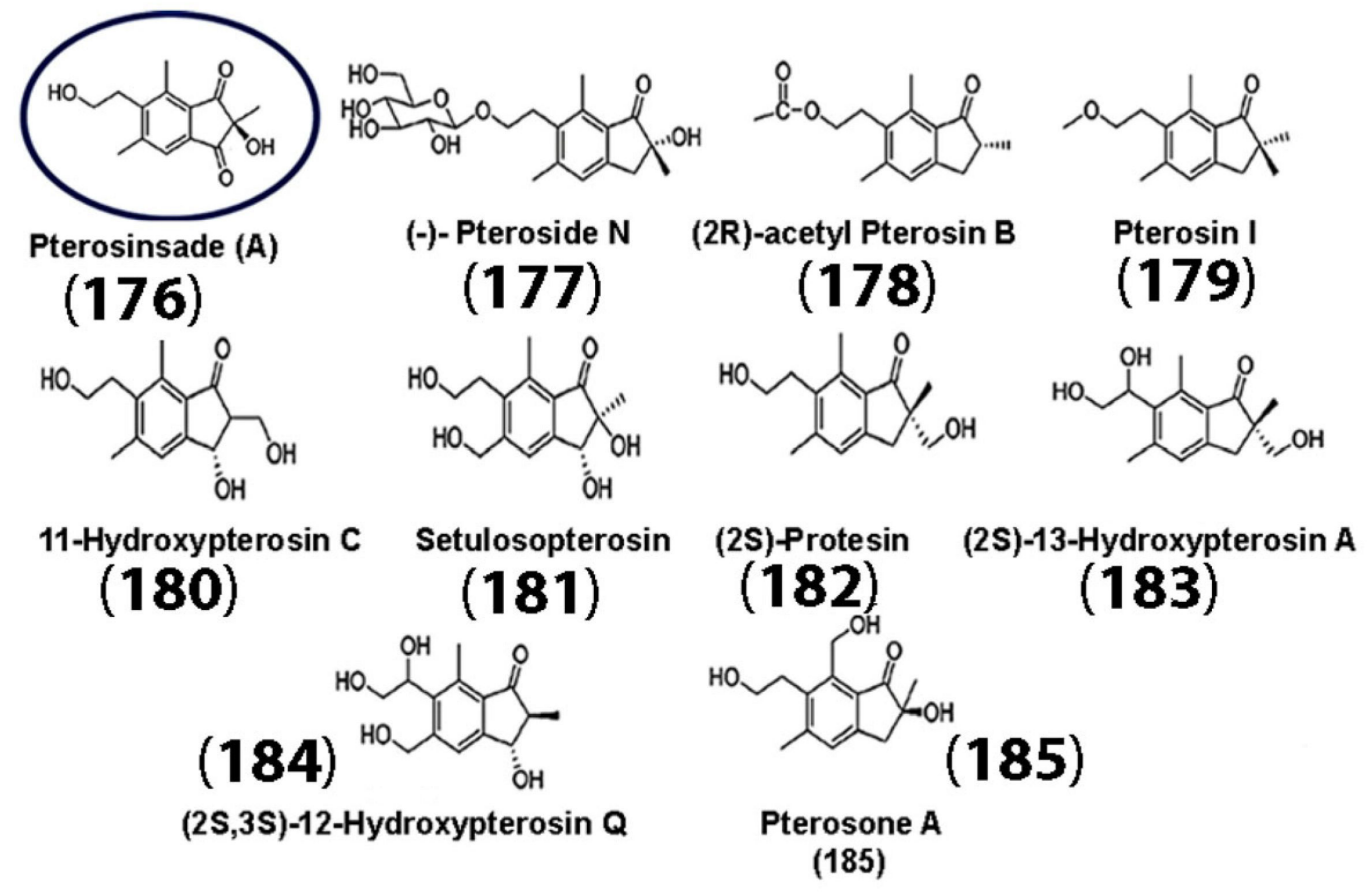
Chemical structures of various sesquiterpenes extracted from *Pteris laeta*. The structure of the newly discovered sesquiterpene PA is circled in blue. (Adapted with permission from the *American Chemical Society* [119]).

The anti-inflammatory and antioxidant properties of sesquiterpenes and their derivatives make them ideal candidates as building blocks for drug development, not only for tumor therapeutics, but also for neurodegenerative diseases. Neuroinflammation and ROS play a key role in neuronal cell damage and exacerbate neurodegenerative disease progression [117]. In Alzheimer’s disease (AD), for instance, misfolded amyloid-β proteins, Aβ (1-40), and Aβ (1-42) aggregate in the brain, forming neurofibrillary tangles that eventually form senile plaques. This is aided by redox-active metal ions, such as copper, iron, or zinc, which can potentially catalyze the formation of ROS when bound to amyloid-β and lead to oxidative damage [118]. Thus, various strategies are being developed to mitigate neuroinflammation and oxidative damage in neurons. In a recent study, the activities of the newly discovered sesquiterpene pterosinsade A (PA) (**176**), derived from *Pteris laeta* (PW) extracts, along with several known sesquiterpenes (compounds (**177**) through (**185**)) (Figure 9) [119] were assessed. The researchers examined the ability of the PW extract and its active constituent PA (**176**) in the prevention of pathological changes that occurred upon Aβ deposition. It was found that PA (**176**) enhanced the survival of APP-overexpressing neuronal cells, as well as their proliferation and differentiation through the Wnt signaling pathway in mice. Furthermore, HT22 (mouse hippocampal neuronal) cells that were pretreated with PW before incubation with Aβ displayed increased cell viability with only 20 and 40 μg/mL of PW.

Additionally, PW extract induced improved levels of indicators of oxidative stress, such as superoxide dismutase (SOD) and glutathione (GSH), and decreased malondialdehyde (MDA). It is well known that Aβ proteins also upregulate pro-apoptotic *Bax* and *Caspase-3* genes and downregulate anti-apoptotic *Bcl-2*, leading to neuronal cell death. However, PW treatment reversed these effects and decreased Aβ-induced apoptosis. Additionally, PA promoted the expression of the Wnt3a and β-catenin proteins, both key proteins for hippocampal neural stem cell (NSC) proliferation and differentiation, which can lead to increased adult hippocampal neurogenesis (AHN). These results are promising, and it is anticipated that further work on the functionalization of PA may potentially lead to the mitigation of Aβ deposition. Furthermore, the researchers also concluded that PA may have the ability to potentially reduce cognitive impairment.

### 5.2. Sesquiterpene Alcohols

Similar to AD, oxidative stress plays a key role in the degeneration of neurons in Parkinson’s disease (PD), causing alterations in the redox potential of neurons, particularly in the substantia nigra region of the brain. This leads to mitochondrial dysfunction, ROS generation, iron overload, and upregulation of genes such as PINK1 and DJ-1, which accelerate PD pathology [120,121]. Preserving the activity of dopaminergic cells and regulation of neurotransmitters are therefore key factors to be considered when developing therapeutics for Parkinson’s disease (PD) [122]. A recent study by Forouzanfar et al. demonstrated that treatment with cedrol (**186**) (Supplementary Information Figure S16), a sesquiterpene alcohol derived from cedar trees, improved SOD and reduced MDA levels in PD-induced mouse models [123]. The same study focused on the cognitive and motor functions of PD-induced mice and found that treatment with cedrol improved results in apomorphine-induced rotational tests, rotarod tests, and other such experiments related to mouse mobility behavior. PD induction in this study was carried out by direct injection of 6-hydroxydopamine (6-OHDA) into the mouse medial forebrain bundle (MFB), which generated ROS and led to mitochondrial impairment in the substantia nigra, as well as the death of dopaminergic neurons. Their results indicate that cedrol can provide neuroprotective effects; however, it was not clear whether dopamine or other neurotransmitters were being modulated in the brain.

In another study, cedrol (**186**) was also found to improve memory in mice AD models that were injected with lipopolysaccharide (LPS) to induce neuroinflammation [124]. It was shown that cedrol (**186**) treatment improved SOD levels and lowered MDA levels compared to untreated mice, further cementing its antioxidant properties. Treatment with cedrol also improved the memory of LPS-induced mice in cognitive tests. Additionally, cedrol was found to reverse the increased acetylcholinesterase (AChE) activity induced by LPS, a promising result indicating that it could be used to improve cognitive dysfunction for AChE regulation of acetylcholine levels in AD patients.

### 5.3. Sesquiterpene Lactones in Neurodegenerative Diseases

**Figure 10:**
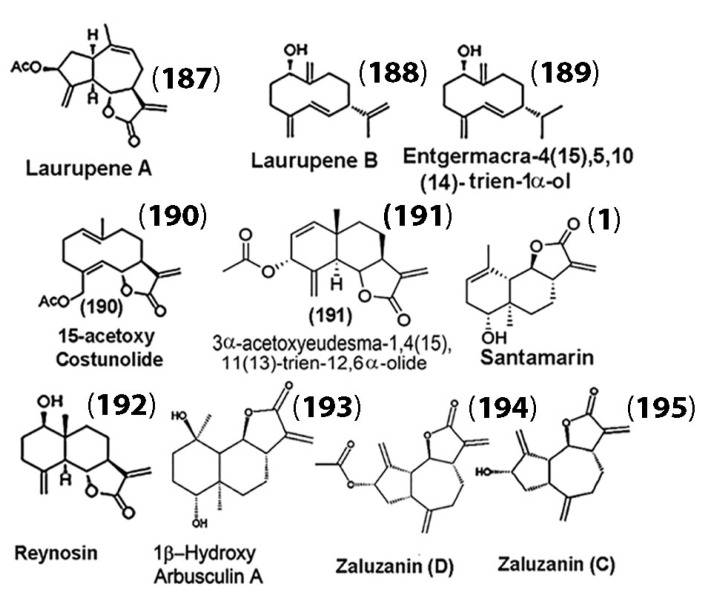
Sesquiterpenes isolated from *L*. *nobilis* for inhibitory studies against glial cell activation. (Adapted with permission from the *American Chemical Society* [125]).

While SLs and their derivatives have been discussed in detail with respect to their anticancer properties in the previous sections, SLs have also been gaining traction for the potential treatment of neurogenerative diseases. For example, Chen and co-workers extracted the SLs laurupene A (**187**) and laurupene B (**188**), along with eight other known analogues (compounds (**189**)–(**195**), (**1**)), from *Laurus nobilis* (Figure 10) [125].

They examined the isolates for possible inhibitory effects on microglial cell activation using murine microglial BV-2 cells. Their results indicated that all the isolates demonstrated inhibitory effects on LPS-induced microglial cell activation. The inhibition levels of microglial activation were correlated with the amount of nitric oxide (NO) production in BV-2 cells. Their results showed that of the isolates, zaluzanin D (**194**) was the most potent against microglial activation. Their results also suggested that the sesquiterpenes derived from *L. nobilis*, particularly 15-acetoxycostunolide (**190**), santamarin (**1**), reynosin (**193**), and zaluzanin D (**195**), which showed strong inhibitory activities of microglial activation, may be considered for further development of drugs for the treatment of neurodegenerative diseases.

**Figure 11:**
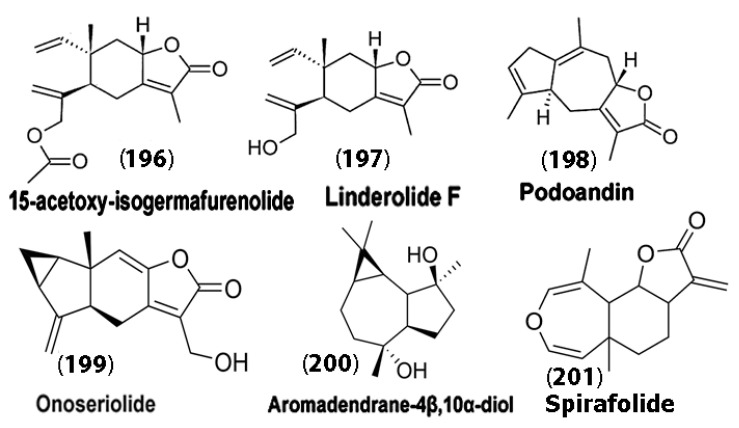
Chemical structures of compounds (**196**)–(**200**) that resulted in an increase in reduced glutathione activity in mice upon treatment. Spirafolide (**201**) derived from *Laurus nobilis* was found to reduce dopamine-induced apoptosis in neuroblastoma cells. Adapted from [126].

Interestingly, sesquiterpene lactones and sesquiterpene alcohols extracted from the leaves of *H. brasiliense* plants have demonstrated strong anti-oxidative, anti-inflammatory, and neuroprotective effects in amyloid beta protein-induced Alzheimer’s disease in mouse models. Specifically, Amoah et al. [126] experimented with SLs, such as IGM-A (15-acetoxy-isogermafurenolide) (**196**), linderolide F (**197**), PDA (podoandin) (**198**), and HDS (onoseriolide) (**199**) and the sesquiterpene alcohol ARD (aromadendrane-4β,10α-diol) (**200**) (Figure 11). Their results demonstrated that ARD, HDS, and PDA showed profound effects, particularly in reducing memory impairment in Alzheimer’s-induced mice after seven days of treatment. Furthermore, an increase in glutathione (GSH) activity was observed. This is particularly promising, as GSH is the primary defense against oxidation in the brain, and GSH levels are decreased in patients with AD, PD, and ALS [127]. The authors attributed the activity of these SLs and the sesquiterpene alcohol to the hydrophobic rigid C1-C5 ring of the guaiane sesquiterpenes of ARD and PDA, which showed the most interactions, while the elemane family compounds like IGM-A showed weaker interactions due to their open chains. It was concluded that PDA, ARD, and HDS may be further developed for potential therapeutics for Alzheimer’s disease.

SLs have also been shown to be potentially applicable in developing treatment options for amyotrophic lateral sclerosis (ALS) disease. In a study conducted by Thau-Habermann and co-workers, PTL (**20**) was shown to suppress the release of TNF-α and thereby mitigate the negative effects of LPS-stimulated microglial cells on motor neurons. Specifically, the group evaluated wild-type and transgenic mouse microglial cells overexpressing the SOD1 mutant G93A and examined specific immunological markers, including TNF-α, iNOS, and IL-1β. Remarkably, it was shown that PTL reversed LPS-induced changes in the reactive state of wild-type and mutant SOD1G93A primary microglial cells [128]. However, TNF-α expression did not cause much changes at lower concentrations of PTL (**20**), but treatment with higher concentrations yielded better results. Overall, their results indicated that in co-cultures of motor neurons and microglial cells, microglia were rescued from LPS activation by the reduced release of TNF-α and cytokine Il-1β, and degradation of motor neuron axon density was reversed compared to untreated neurons. Additionally, PTL (**20**) was shown to indirectly affect astrocytes in co-cultures with microglia by modulating the secretome of the microglia. Interestingly, PTL (**20**) showed no effects on monocultures of motor neurons. These results were extremely promising given the impact of glial cells and motor neurons in ALS and other neurodegenerative diseases. Overall, the authors concluded that PTL (**20**) can exert neuroprotective effects and may be developed as a potential drug candidate for ALS.

The SL spirafolide (**201**) (structure shown in Figure 11) derived from bay leaves (*Laurus nobilis*) has also been shown to demonstrate neuroprotective effects by reducing dopamine-induced apoptosis in neuroblastoma SH-SY5Y cells. Mechanistically, it was shown that spirafolide (**201**) significantly lowered intracellular ROS and, therefore, may be able to contribute toward developing drugs for the treatment of neurodegenerative diseases [129].

More recently, SLs have also been conjugated with neurotransmitters such as serotonin to develop drug candidates for the potential treatment of neurodegenerative diseases. For instance, Neganova and co-workers conjugated sesquiterpene lactones with serotonin and examined their potential as treatments for Alzheimer’s disease [130]. They developed four different methods for the conjugation of serotonin with three natural SLs (isoalantolactone (**25**), alantolactone (**24**), and epoxy-isoalantolactone (**140**)), resulting in three new conjugates. Serotonin–amine generation in situ was utilized to avoid premature degradation of serotonin when converted to a base for Michael addition. The serotonin–sesquiterpene conjugates were assayed in vitro for antioxidant properties, and the results indicated that the serotonin conjugates decreased the production of MDA. Consistent with previous studies of sesquiterpene lactones, these conjugates exhibited neuroprotective anti-inflammatory and antioxidant effects. The serotonin conjugates also decreased Ca^2+^-induced mitochondrial swelling and uptake, as well as the stimulation of electron transport chain complexes, which can combat the negative impact of Aβ on cytochrome c oxidase and electron transport chain inhibition. Furthermore, the serotonin–SL conjugates were studied for their direct effects on Aβ production and aggregation in mice. Remarkably, all three conjugates blocked beta-secretase 1 (BACE1) activity. Additionally, when incubated with each conjugate respectively, Aβ (1-42) formation was reduced, as indicated by decreased fluorescence in thioflavin-T tests, indicating that the serotonin derivatives led to conformational changes in the beta-sheets of Aβ and mitigated aggregation. Thus, it appears that the serotonin–SL conjugates can effectively mitigate both the production and aggregation of Aβ. In vivo studies in Alzheimer’s-induced mice showed that, in particular, the serotonin conjugate with isoalantolactone was found to reduce cognitive dysfunction and memory loss in mice. Additionally, the mice were sacrificed, and brain samples displayed that GSH levels increased and MDA levels decreased when treated with the serotonin–isoalantolactone conjugate, again suggesting that the serotonin conjugates have strong antioxidant properties and protective effects on mitochondria in AD models. These conjugates prove especially promising, as they seem to hit multiple targets desirable for AD treatment. Furthermore, these studies provide the groundwork for the potential development of sesquiterpene conjugates with other neurotransmitters and for investigating the ability of such conjugates in neurodegenerative diseases.

## 6. Pharmacokinetics and Toxicity Studies of Sesquiterpenes and Their Derivatives

ADME (absorption, distribution, metabolism, and excretion) studies play a key role in assessing the likelihood and efficacy of drug molecules. While extensive pharmacokinetics and tissue distribution studies have yet to be carried out with some of the newly designed synthetic sesquiterpene-derived drug molecules described in this review, several studies have been conducted to assess the pharmacokinetic properties of naturally derived sesquiterpenes and their derivatives. For example, Wu and co-workers recently showed that sesquiterpene glycosides, such as dendronobiloside A (**202**), dendronobiloside C (**203**), and dendronobiloside D (**204**), were rapidly absorbed at low plasma concentrations and were also rapidly eliminated. However, dendronobilosides E (**205**) (Supplementary Information Figure S17) and dendroside G were absorbed at higher plasma concentrations but were also excreted rapidly. Furthermore, tissue distribution studies in rats revealed that dendronobiloside A, C, and D were found in a number of organs, including the liver, spleen, lungs, kidneys, heart, stomach, large and small intestines, pancreas, and thymus. Interestingly, these were not detected in the brain. In contrast, dendronobiloside E, D, and G were also found in the brain in addition to the other organs. Furthermore, dendronobiloside E, D, and G showed longer half-lives [131]. The results of their study open new doors for future work related to further modification of these molecules to enhance pharmacokinetic and pharmacodynamic properties. Additionally, the inhibitory activity of (**202**) and (**203**) against α-glucosidase has been tested. Results showed that the compounds inhibited the activity of the enzyme, with IC_50_ values of 299.7 ± 2.4 μg/mL and 537.8 ± 2.3 μg/mL, respectively [132]. While these values are relatively high, the compounds may be chemically modified to enhance their activity, given that α-glucosidase inhibitors have been found to reduce the risk of cancer in diabetic patients [133]. In another key study conducted by Xi and co-workers, the drug ACT001 (dimethylaminomicheliolide, DMAMCL) (**206**), a synthetic derivative of the guaianolide SL micheliolide (**207**), which is currently undergoing phase II clinical trials [134] and demonstrated promising results against glioblastoma in Phase I clinical trials, has been shown to penetrate the blood–brain barrier (BBB) and accrue in the brain at higher concentrations than in plasma in rats upon oral administration of the drug [135]. Additional ADME studies revealed that the oral bioavailability of the drug was found to be 50.82%, and that within a half hour of oral administration of the drug in Sprague Dawley rats, ACT001 was widely distributed in tissues, including the liver, spleen, lung, kidney, brain, heart, stomach, duodenum, testicles, ovaries, and muscles; however, after ten hours, the levels of the drug in these tissues declined. Furthermore, the drug was found to metabolize into five different metabolites, with micheliolide (**207**) being the key metabolite found [136]. In other studies, ACT001 was shown to enhance the effects of the chemotherapeutic drug cisplatin [137] and therefore appears to be an extremely promising drug. In general, ACT001 has been found to demonstrate cytotoxicity against a variety of cancer cell lines, including non-small cell lung cancer cell lines (H1975, IC_50_ =14.4 μM; H1703, IC_50_ = 9.21 μM) [138] and glioma cells (U-251, IC_50_ = (75.4 ± 10.9) μM; SF-126, IC_50_ = (25.2 ± 7.5) μM; U-118MG (18.7 ± 1.8) μM) [139], among others.

In vitro and in vivo studies with alantolactone (**24**) and isoalantolactone (**25**) have also been conducted and showed low oral bioavailability based on studies conducted in rats, and those results revealed that the liver was the primary metabolic organ that led to the formation of five metabolites in the liver microsomes for alantolactone and four metabolites for isoalantolactone [140]. Acute toxicity studies with the alantolactone derivative AL-04 (**208**) (IC_50_ = 17.81 ± 1.86 µM for the inhibition of nitric oxide (NO) production in LPS-stimulated RAW 264.7 cells) showed that it exerts strong anti-inflammatory effects, and it was found to be safe at a high dose of 2000 mg/kg, with no indications of toxicity or alterations in biochemical and hematological features in BALB/c mice [141]. The chemical structures of compounds (**206**) through (**208**) are shown in Supplementary Information Figure S18. Additional studies have also shown that thiolated derivatives of alantolactone (**24**) demonstrate strong anti-inflammatory properties [142], and further clinical trials and toxicity studies are necessary for developing these derivatives into therapeutics. Subacute toxicity studies with *T. Parthenium* ethanol extracts, which are rich in PTL (**20**), have also been conducted in BALB/c mice. Results showed that while serum and hematological parameters did not show a major change, the histopathology of the liver showed steatosis, indicating liver toxicity [143]. Thus, while parthenolide (**20**) is a very promising SL, like many of the sesquiterpenes discussed here, it is likely that developing more bioavailable conjugates with reduced toxicity would aid in the development of therapeutics.

## 7. Conclusions and Future Directions

Sesquiterpenes and their structurally varied derivatives continue to emerge as powerful bioactive scaffolds with multifaceted therapeutic potential. Obstacles blocking the derivatization and analogue development of sesquiterpenes and SLs are being tackled through new methodologies and reactions that increase the bioavailability and tunability of these compounds. The wide range of chemical modifications, ranging from triazole and arylidene functionalization to conjugation with chemotherapeutics and neurotransmitters, has significantly enhanced their pharmacological profiles, targeting ability, and bioavailability. In cancer research, sesquiterpenes demonstrate robust cytotoxicity via multiple mechanisms, including NF-κB and TrxR1 inhibition, mitochondrial ROS induction, and metabolic reprogramming. The adjustable properties, such as size, specificity, and antioxidative or anti-inflammatory properties, depending on the SL used for conjugation or derivatization, provide the potential for higher specificity. Being able to select specific targets such as glycolytic, COX-2, prostaglandin, or other pathways to inhibit tumor cell proliferation can allow for the avoidance of unwanted side effects specific to different types of cancers. Additionally, the ability of conjugates of SLs to suppress growth factors and reverse drug resistance by reversing drug expulsion pathways can make these treatments even more impactful. Nanoformulations of sesquiterpene derivatives can potentially exhibit higher efficacy due to their tunability. Peptide-based nanopartherapeutics with sesquiterpenes should be further developed in order to potentially increase targeting ability.

Likewise, the role of sesquiterpene derivatives in combating neurodegenerative diseases through antioxidant, anti-inflammatory, and amyloid-modulating pathways is increasingly being recognized. The ability of sesquiterpenes and SL conjugates to promote cell differentiation and even neurogenesis while combating neuroinflammation and glial cell activation makes them promising candidates for drug development in this area. It has been shown that sesquiterpenes possess neuroprotective features, such as strong anti-inflammatory and antioxidant properties, which allow them to combat the pathologies of PD, AD, and ALS. Sesquiterpenes isolated from natural extracts alone can mitigate glial cell activation and even indirectly protect motor neurons outside the CNS. There is strong potential for developing functional derivatives for further tunability of conjugates in this field. Even more promising are the sesquiterpene–neurotransmitter conjugates, which show benefits in multiple aspects for developing a therapeutic for AD. Additional neurotransmitter derivatives could prove to have stronger modulating effects on the neuronal cell environment. There are many possible conjugates and derivatives of sesquiterpenes that may be designed, synthesized, or transferred over from anticancer studies to examine their direct effects on proteins such as Aβ and α-synuclein. Most of the studies examined solidified the neuroprotective effects of sesquiterpenes, but there needs to be a major step forward in using them for the development of prodrugs or drugs to combat neurodegenerative diseases. Additionally, nanoparticles conjugated with SLs or sesquiterpenes could be used to enhance the specificity of drugs for Aβ, Lewy bodies, or other targets, as well as allow blood–brain barrier penetration.

Despite these advances, several challenges remain. Many sesquiterpene-based compounds and the newly developed conjugates lack clinical validation due to limited pharmacokinetic data, poor solubility, or toxicity profiles. Future directions should prioritize structure–activity relationship (SAR) studies, in vitro and in vivo evaluations, and translational research that conduits synthetic chemistry, nanotechnology, biochemistry, and molecular biology. Moreover, integrating sesquiterpenes into multifunctional drug platforms, especially through self-assembling nanocarriers or drug conjugates, may address multidrug resistance and blood–brain barrier permeability. Although this review has primarily focused on the anticancer and anti-neurodegenerative potential of sesquiterpene derivatives, many conjugates have been and may be developed that can exploit the antimicrobial activities of these natural products. Altogether, this review underscores the urgent need for collaborative, interdisciplinary research to open up the full therapeutic potential of sesquiterpenes and their many derivatives. With continued innovation, these compounds are poised to play a transformative role in next-generation treatments for cancer and neurodegenerative disorders.

## Figures and Tables

**Table 1:** Summary of the bioactivities of naturally occurring selected sesquiterpenes and their derivatives.

Sesquiterpene/SL/S-Alcohol/Esters	IC_50_ Values (μM)	Cancer Cell Lines Targeted	In Vitro Studies	In Vivo Studies	Ref. #
(**20**)	6.07 ± 0.45	non-small cell lung cancer GLC-82 cells	MTT assays proved cytotoxicity. Annexin-FITC apoptosis assays proved apoptosis. PTL targeted B-Raf and inhibited the MAPK-ERK pathway. Inhibition of STAT3 phosphorylation was observed using Western blot studies. Cell motility was reduced, as shown by scratch assay.	Human lung tumor tissues were examined. Immunohistochemistry was used to examine expression of B-Raf and c-Myc proteins in NSLC samples.	[41]
	15.38 ± 1.13	A549	MTT assays proved cytotoxicity	-	[41]
	15.36	PC-9	“ ”	-	[41]
	9.88 ± 0.09	H1650	“ ”	-	[41]
	12.37 ± 1.21	H1299	“ ”	-	[41]
(**23**)	2.2 5.9 3.5 4.7 ± 0.1 3.3 ± 0.3	HepG2 MCF-7 A549 NCI-H460 NCI-H460/R (MDR cancer cell lines with P-gp over-expression)	MTT assay was conducted to study cytotoxicity, Tunel assay was performed to confirm apoptosis. MTT assay was conducted to confirm cytotoxicity.	-	[37,38]
(**24**)	0.15 ± 0.09/ 2.75 ± 0.64	K562/A02 (Drug resistant cell line) K562	Cytotoxicity was assayed by MTT to confirm the inhibition of cell proliferation. “ ”	-	[40]
	0.027 ± 0.02 3.26 ± 0.88	HL60 Adriamycin Resistant cell line HL60	Cytotoxicity was assayed by MTT. Induction of apoptosis and differentiation of AML stem and progenitor cells were observed. Cytotoxicity was attributed to apoptosis by the suppression of NF-kB and its downstream proteins. p65, XIAP, FLIP, Bcl-2, Bax, and the cleavage of PARP, caspase-3, and caspase-9 were detected by Western blots in primary CD34^+^ cells, HL60, and KG1a cells	(**24**) was converted to its prodrug, DMA-ATL (water soluble). Toxicity was first evaluated in normal Kunming mice. Then, tumors were induced in mice by injecting KG1 cells. Mice were treated with the pro-drug. Tumor sizes were significantly suppressed.	[40]
	2.17 ± 0.72	THP-1	Cytotoxicity was confirmed through MTT assay.	-	[40]
	2.75 ± 0.65	KG1a	“ ”	-	[40]
	35.99 ± 5.55	GLC-82	“ ”	-	[41]
	32.89 ± 5.60	A549	“ ”	-	[41]
(**25**)	53.4	HepG2 cells	Cytotoxicity was evaluated through MTT assays. Mechanistically, apoptosis was proven, and higher expression of Bax and caspases 3, 9, and 8 and lower expression of Bcl-2 proteins was observed. Ras/Raf/MEK signaling was suppressed. Flow cytometry showed induction of ROS, and cell migration and invasion assays showed inhibition.	-	[42]
	10	HEC-1-B endometrial cancer cells	Cytotoxicity was evaluated through MTT assays. Induction of ROS mediated apoptosis, and inhibition of the MEK/ERK signaling pathway was proven through flow cytometry, fluorescence staining and Western blots, which showed reduction in the expression of ERK and MEK by inhibiting their phosphorylation. Cell migration was inhibited.	-	[43]
	17.09 ± 1.68	GLC-82	Cytotoxicity was confirmed through MTT assay.	-	[41]
	17.12 ± 1.62	A549	Cytotoxicity was confirmed through MTT assay.	-	[41]
(**28**)	81.45 ± 3.8	A549	MTT cytotoxicity assay and apoptosis assay.	-	[44]
	10.82 ± 0.5	HeLa	“ ”	-	[44]
	62.58 ± 5.1	HepG2	MTT assay and Annexin-V flow cytometry showed apoptosis. Cell cycle analysis showed G2/M phase cell cycle arrest.	-	[44]
(**29**)	13.61 ± 0.25	A549	Cytotoxicity was proven through MTT assay, and Annexin-FITC assays proved apoptosis.	-	[44]
	3.81 ± 0.36	HeLa	“ ”	-	[44]
	15.47 ± 1.69	HepG2	MTT assay and Annexin-V flow cytometry proved apoptosis. Cell cycle analysis showed G2/M phase cell cycle arrest.	-	[44]
(**32**)	19.7	PC-3	WST-1 cytotoxicity assay.	“	[47]
(**34**)	0.8 100.57 39.92	A431 MDA-MB-231-Luc HCT116	MTT assay confirmed cytotoxicity. Costunolide activated p38 and c-Jun N-terminal kinase pathways and suppressed ERK, STAT3, NFκ-B, and Akt pathways. MTT assay confirmed cytotoxicity. Apoptosis was confirmed through assessment of BAX and Bcl2 protein level measurements.	- Xenograft mice models were studied and showed decreased tumor growth. Significant necrotic and apoptotic cells were observed when given alongside the doxorubicin.	[45,46]
(**35**)	3.8 ± 0.1/15.1 ± 0.4	NCI-H460 NCI-H460R	MTT assay was conducted to study cytotoxicity.	-	[38]
(**36**)	8.2 ± 0.1/ 4.5 ± 0.1	NCI-H460 NCI-H460/R	MTT assay was conducted to confirm cytotoxicity.	-	[38]
(**37**)	7.5 μg/mL 4.0 μg/mL 1.9 μg/mL 3.4 μg/mL	HCT 116 K562 T47D KB	MTT assay showed cytotoxicity. ROS was upregulated in tumor cells upon treatment. Both mitochondrial and receptor mediated apoptosis were observed. Autophagy and decrease in NF-κB and IκBα was observed. Downregulation of MMP-2 and MMP-9 was observed.	-	[39]

**Table 3:** Summary of the bioactivity of selected guaiazulene and synthesized derivatives.

Compound	IC_50_ (μM)	Cell Line	In Vitro Studies	In Vivo Studies	Ref. #
(**108**)	110 160 120	HSG HSC-2 HSC-3 (Human oral cancer cell lines)	MTT assay was conducted to confirm cytotoxicity. DNA fragmentation analysis and induction of caspase 3 activity was studied.	-	[70]
(**109**)	8.66 8.35	K562 MDA-MB-231	MTT assay for cytotoxicity; PPAR-γ signaling assay to determine the agonistic effects of the derivatives.	Anti-inflammatory activity was tested in zebrafish models.	[71]
(**110**)	18.8 >30	K562 MDA-MB-231	MTT assay for cytotoxicity; PPAR-γ signaling assay to determine the agonistic effects of the derivatives.	Anti-inflammatory activity was tested in zebrafish models.	[71]
(**111**)	5.21 30	K562 MDA-MB-231	MTT assay for cytotoxicity; PPAR-γ signaling assay to determine the agonistic effects of the derivatives.	Anti-inflammatory activity was tested in zebrafish models.	[71]
(**112**)	35 48 47 38	Ca9-22 HSC-2 HSC-3 HSC-4	MTT assay for cytotoxicity studies were conducted. DNA fragmentation and caspase activation assays were conducted to determine apoptosis. Antioxidant assays were conducted to determine free-radical scavenging ability.	-	[72]

**Table 4:** Summary of the bioactivity of selected drug conjugates with Sesquiterpenes.

Chemotherapeutic Drug Conjugate with SL	IC_50_ (μM)	Cancer Cell Line	In Vitro Assay/Mechanism	In Vivo	Ref. #
5-FU conjugates with PTL (**135**)	2.67 3.24	Bel-7402 (human hepatocellular carcinoma cells) Bel-7402/5-FU (5-FU resistant human hepatocellular carcinoma cells)	MTT assay proved inhibition of cell proliferation. Apoptosis assay confirmed its induction. Nuclear morphology changes were observed.	--	[87]
(**136**)	2.73 2.90	Bel-7402 Bel-7402/5-FU	MTT assay proved inhibition of cell proliferation. Apoptosis assay confirmed its induction. Nuclear morphology changes were observed.		[86]
(**137**)	4.07 4.34	Bel-7402 Bel-7402/5-FU 5-FU	MTT assay proved inhibition of cell proliferation. Apoptosis assay confirmed its induction. Nuclear morphology changes were observed.		[86]
(**138**)	3.98 4.27	Bel-7402 (human hepatocellular carcinoma cells) Bel-7402/5-FU	MTT assay proved inhibition of cell proliferation. Apoptosis assay confirmed its induction. Nuclear morphology changes were observed.		[86]
(**144**)	0.75 0.04 0.73 2.48	A549 HCT116 RD MCF7	MTT assay proved inhibition of cell proliferation.	Cardiotoxicity lowered in rats	[88]
(**145**)	1.38 0.41 1.16 4.13	A549 HCT116 RD MCF7	MTT assay proved inhibition of cell proliferation.	Cardiotoxicity lowered in rats	[88]

**Table 5:** Summary of the bioactivity of naturally occurring selected sesquiterpene quinones/quinols and their derivatives.

Compound	IC_50_ (μM)	Cell Line	Assays and Mechanism	In Vivo Studies	Ref. #
(**148**)	2.8 >10 >10 >10	U937 A549 HeLa HepG2	CCK-8 cytotoxicity assay proved inhibition of cell proliferation; cyclin-dependent kinase (CDK2) binding assays were also conducted using SPR.	-	[94]
(**149**)	1.5 >10 8.6 6.7	U937 A549 HeLa HepG2	CCK-8 cytotoxicity assay proved inhibition of cell proliferation; CDK2 binding assays were also conducted using SPR.	-	[94]
(**150**)	0.60 >10 5.4 3.5	U937 A549 HeLa HepG2	CCK-8 cytotoxicity assay proved inhibition of cell proliferation; CDK2 binding assays were also conducted using SPR.	-	[94]
(**155**)	2.81	NCI-H929 cells	MTT cytotoxicity assay proved inhibition of cell proliferation; NF-κB inhibition was proven by luciferase assays.	-	[95]
(**156**)	5.45	NCI-H929 cells	MTT cytotoxicity assay proved inhibition of cell proliferation; NF-κB inhibition was proven by luciferase assays.	-	[95]
(**157**)	2.77	NCI-H929 cells	MTT cytotoxicity assay proved inhibition of cell proliferation; NF-κB inhibition was proven by luciferase assays.	-	[95]
(**160**)	3.2	NCI-H929 cells	MTT cytotoxicity assay proved inhibition of cell proliferation; NF-κB inhibition was proven by luciferase assays.	-	[95]
(**154**)	14.9	NCI-H929 cells	MTT cytotoxicity assay proved inhibition of cell proliferation; NF-κB inhibition was proven by luciferase assays.	-	[95]
(**161**)	8.9 5.9 8.6	A549 MCF7 HeLa	WST-8 cytotoxicity assay was carried out to prove inhibition of cell proliferation.	-	[96]
(**162**)	39.9 - - -	HeLa A549 MDA231 Hepatoma (QGY7703)	Cytotoxicity was confirmed by MTT assay. Protein tyrosine phosphatase B inhibition was also observed.	-	[97]
(**163**)	28.8 21.4 11.6 28.1	HeLa A549 MDA231 Hepatoma (QGY 7703)	Cytotoxicity was confirmed by MTT assay. Protein tyrosine phosphatase B inhibition was also observed.	-	[97]
(**164**)	2.63	LPS treated RAW 264.7 macrophage cells	Anti-inflammatory activity was exhibited through inhibition of the NF-κB-NLRP3 axis. NF-κB nuclear translocation was observed.	-	[98]

## Data Availability

No new data were generated in this review article. All studies and data reported are available publicly from the references cited.

## Supplementary Materials

The following supporting information can be downloaded at: https://www.mdpi.com/article/10.3390/molecules30214302/s1, Figure S1: Chemical structures of naturally occurring sesquiterpene lactones (**28**) through (**37**); Figure S2: Chemical structure of psilostachyin B (**39**); Figure S3: Chemical structure of ferutinin analogue jaesckeanadiol-3-p-hydroxyphenylpropanoate (**46**); Figure S4: Chemical structures of SLs ludartin (**47**) and melampomagnolide (**48**); as well as widdaranal based sesquiterpenes (**52**), (**55**); its peroxide derivative (**51**) and isocurparenal (**56**). Chemical structure of 1,2,3 triazole derivative of β-himachalene (**50**) is also shown. (Adapted from Reference [55]); Figure S5: Chemical structures of 1-O-acetylbritannilactone and some of its azole derivatives that have been reported. (imidazole derivative (62a); 1,2,4 triazole derivative (62b) and 1,2,3 triazole derivative (62c). (Adapted from reference [58]); Figure S6: Chemical structures of 1-O-acetylbritannilactone and some of its azole derivatives that have been reported. (imidazole derivative (62a); 1,2,4 triazole derivative (62b) and 1,2,3 triazole derivative (62c). (Adapted from reference [58]).; Figure S7: Chemical structures of synthesized 3,5-bis(arylidene)-4-piperidone derivatives of dehydrocostus lactone (**63**). (Adapted from Reference [63]); Figure S8: Chemical structures of (-)-santonin and α-desmotroposantonin; Figure S9: Amino acid ester trifluoroacetate derivative (**131**) of scaberol C (**122**) (Adapted from Reference [78]); Figure S10: Sesquiterpene structures (**139-143**) that have been utilized for the synthesis of chemotherapeutic drugs; Figure S11: Chemical structures of chemotherapeutic drug-SL conjugates (i–iv) show some of the 5-fluorouracil conjugates with parthenolide (**135-138**); (v and vi) show alantolactone and isoalantolactone conjugates with daunorubicin (**144** and **145**); Figure S12: Chemical structures of xanthatin (**146**) and beta-caryophyllene oxide (**147**); Figure S13: Chemical structures of additional sesquiterpene quinones seriniquinone (**165**) and avarone (**166**); Figure S14: Chemical structures of guaiane-type sesquiterpene lactones aguerin (**167**); cynaropicrin (**168**) and grosheimin (**169**) used in nanoformulations with lithocholic acid; Figure S15: Chemical structures of sesquiterpenes and some of their modified derivatives that have undergone clinical trials; Figure S16: Chemical structure of sesquiterpene alcohol Cedrol (**186**); Figure S17: Chemical structures of dendronobilosides A, C, D and E (compounds (**202**) through (**205**)); Figure S18: Chemical structure of michelolide derivative ACT001 (**206**); micheliolide (**207**) and thiolated derivative of alantolactone (AL-04) (**208**).
