# Supplementary material for: Advances in Naturally and Synthetically Derived Bioactive Sesquiterpenes and Their Derivatives: Applications in Targeting Cancer and Neurodegenerative Diseases
Source: Molecules. 2025 Nov 5;30(21):4302. doi: 10.3390/molecules30214302 (PMC12609730; doi:10.3390/molecules30214302)
Supplement: Supplementary file 1 [file molecules-30-04302-s001.zip › molecules-3922046-supplementary.pdf]

Supplementary Information

Advances in Naturally and Synthetically Derived Bioactive Sesquiterpenes and their Derivatives: Applications in Targeting Cancer and Neurodegenerative Diseases

L.R. Cutter, A. R. Ren and I. A. Banerjee*

Department of Chemistry and Biochemistry, Fordham University, 441 East Fordham Road, Bronx, New York 10458, USA

In this review, we have endeavored to provide a summary of the literature over the period of past 15 years, with special emphasis on work done over the past decade, particularly for the development of novel chemically functionalized derivatives of sesquiterpenes and their formulations that may be potentially developed for treatment against cancer and neurodegenerative diseases. The literature search strategy involved using databases such as SCOPUS, SciFinder, PubMed, Google Scholar, ACS; RSC; Wiley and ScienceDirect databases. For clinical trials, we https://clinicaltrials.gov and the Cochrane database (Cochranelibrary.com). Keywords included toxicity of sesquiterpenes, chemical functionalization of sesquiterpenes; derivatives of sesquiterpenes; cancer therapeutics and sesquiterpenes; anti-inflammatory pathways, tumor cell inhibition pathways and sesquiterpenes; sesquiterpenes and essential oils; plant derived and marine derived sesquiterpenes; sesquiterpene derivatives and neurodegenerative diseases as well as specific sesquiterpenes and their applications in cancer treatment and Alzheimer's and Parkinson's diseases. In general, articles published in English language and after the year 2010 were selected for most of the work, unless a historical precedent was required. The major criteria involved examining the overall progress in development of therapeutics in relation to sesquiterpenes and their chemical functionalization, and derivatization as well as nanoformulations for cancer and neurodegenerative disease treatment. A total of 134 articles were selected in this review with several others that we also did a thorough analysis of.

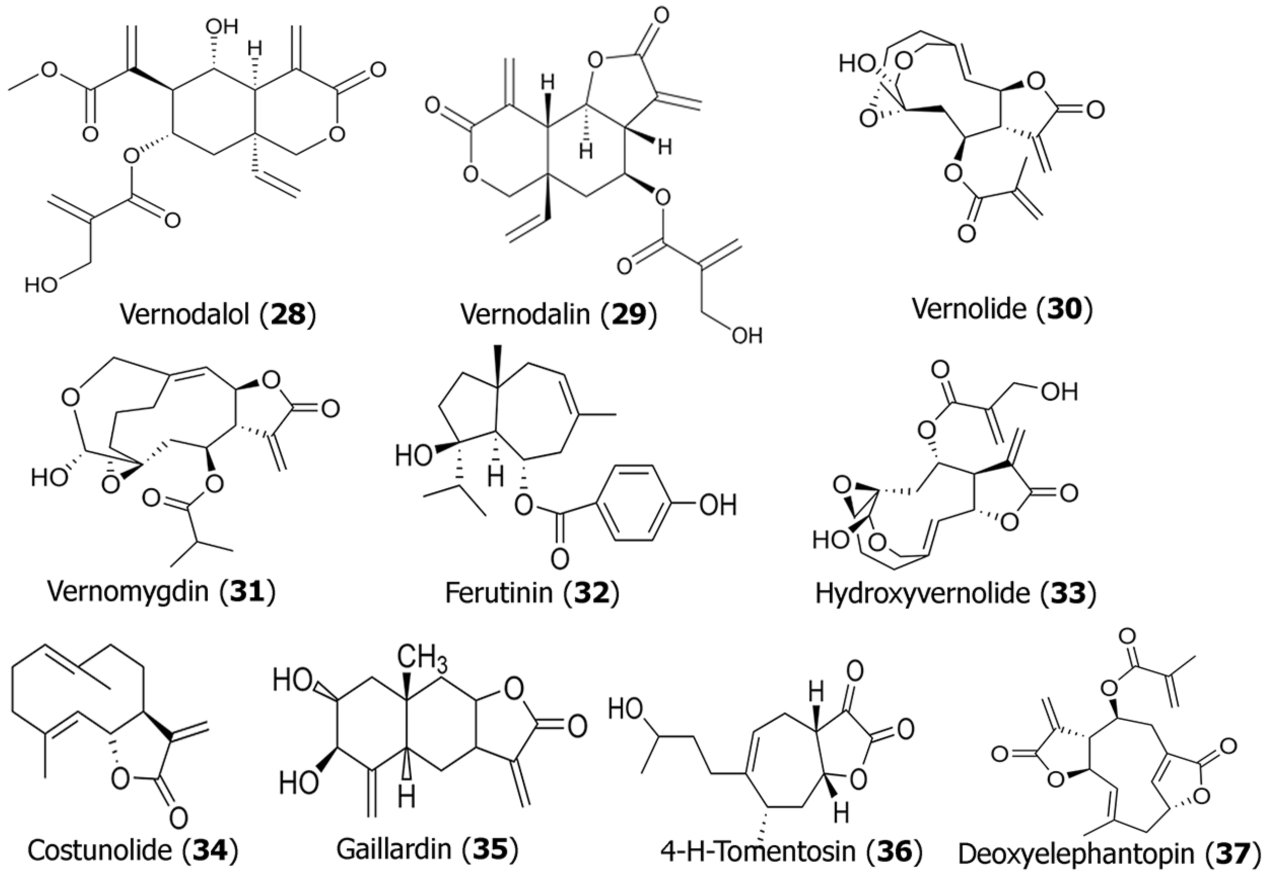

Figure S1. Chemical structures of naturally occurring sesquiterpene lactones (**28**) through (**37**).

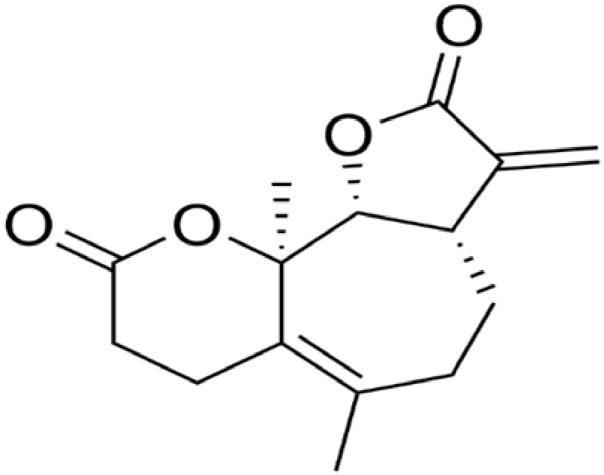

Psi B (**39**)

Figure S2. Chemical structure of psilostachyin B (**39**)

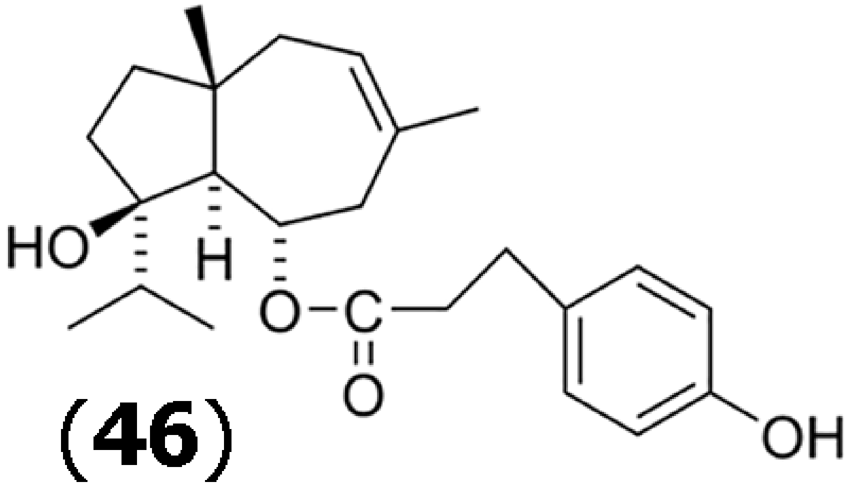

(46)

Figure S3. Chemical structure of ferutinin analogue jaesckeanadiol-3-p-hydroxyphenylpropanoate (**46**)

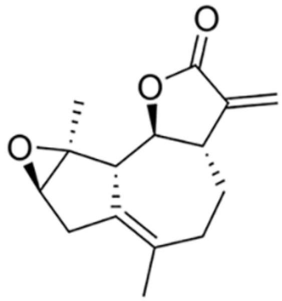

Ludartin **(47)**

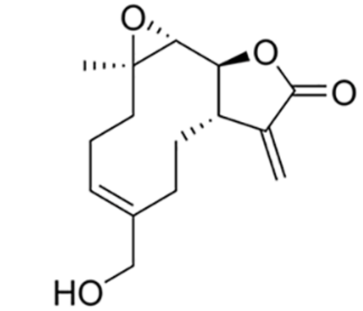

Melampomagnolide B **(48)**

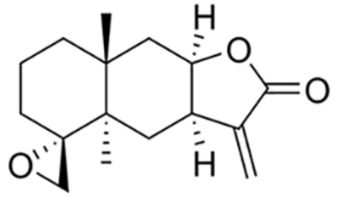

4,15-epoxy isoalantolactone **(49)**

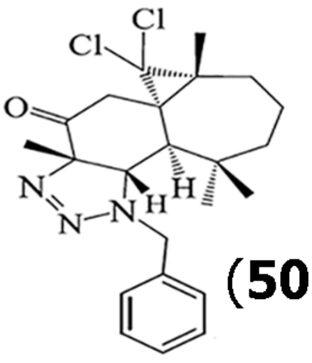

(50)

1,2,3 Triazole derivative of β-himachalene

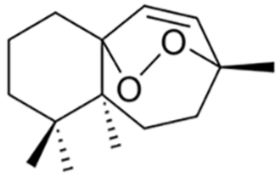

Widdarol peroxide **(51)**

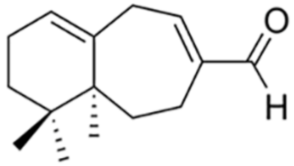

Widdaranal C **(52)**

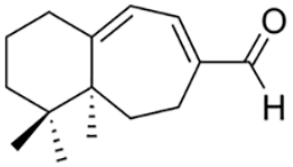

Widdaranal B **(55)**

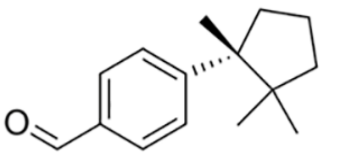

Isocuparenal **(56)**

Figure S4. Chemical structures of SLs ludartin (**47**) and melampomagnolide (**48**); as well as widdaranal based sesquiterpenes (**52**), (**55**); its peroxide derivative (**51**) and isocurparenal (**56**). Chemical structure of 1,2,3 triazole derivative of β-himachalene (**50**) is also shown. (Adapted from Reference 55).

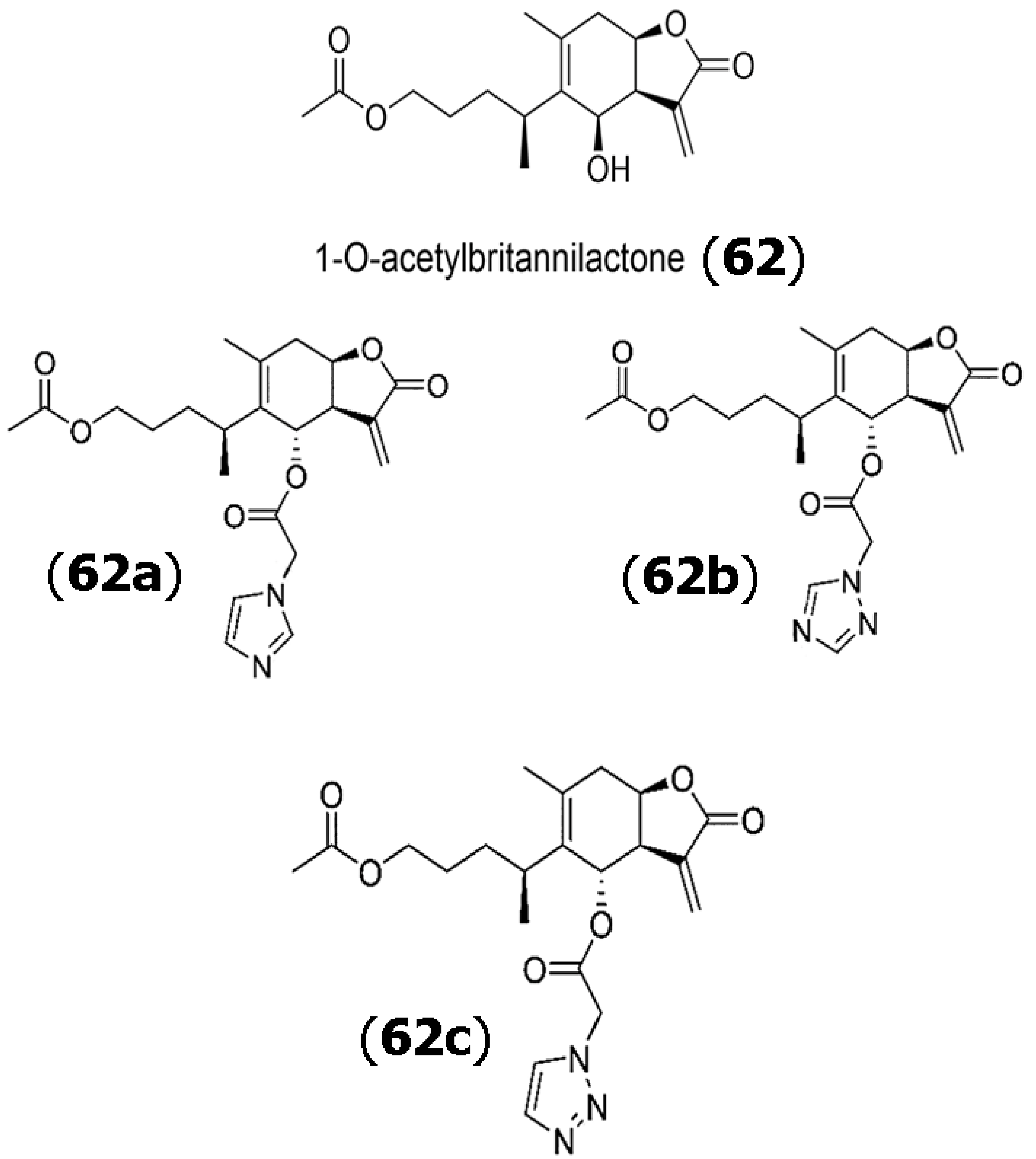

Figure S5. Chemical structures of 1-O-acetylbritannilactone and some of its azole derivatives that have been reported. (imidazole derivative (62a); 1,2,4 triazole derivative (62b) and 1,2,3 triazole derivative (62c). (Adapted from reference 58).

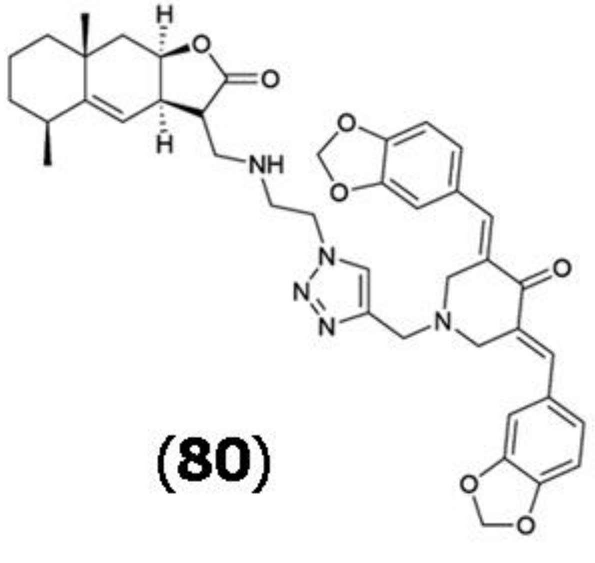

(80)

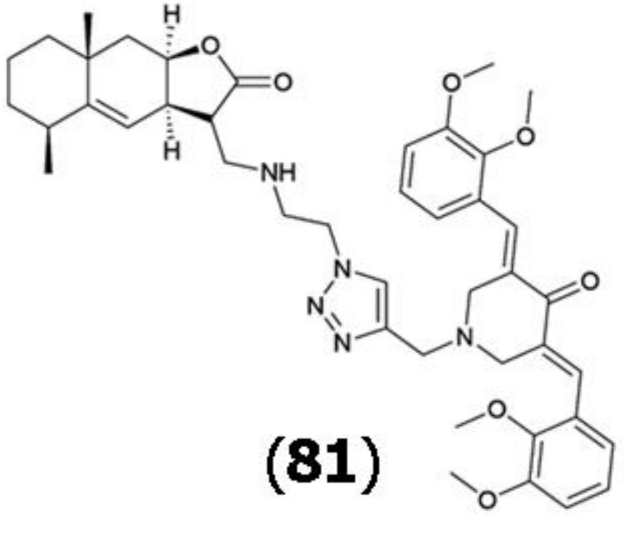

(81)

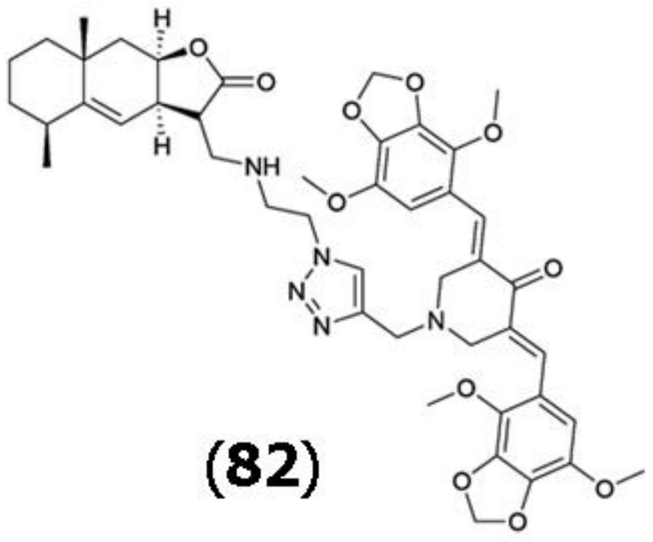

(82)

Figure S6. Chemical structures of 1-O-acetylbritannilactone and some of its azole derivatives that have been reported. (imidazole derivative (62a); 1,2,4 triazole derivative (62b) and 1,2,3 triazole derivative (62c). (Adapted from reference 58).

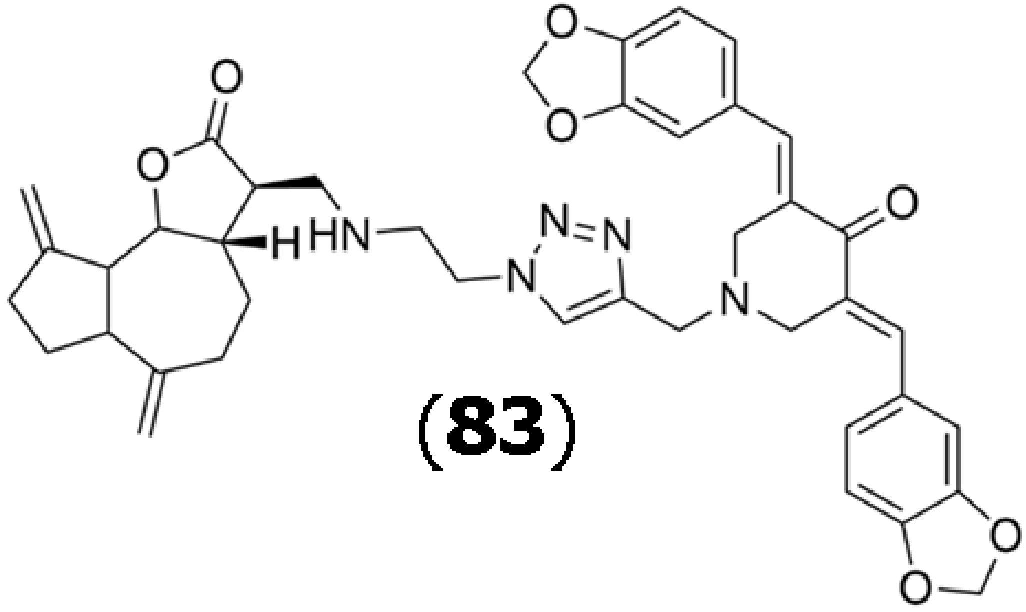

(83)

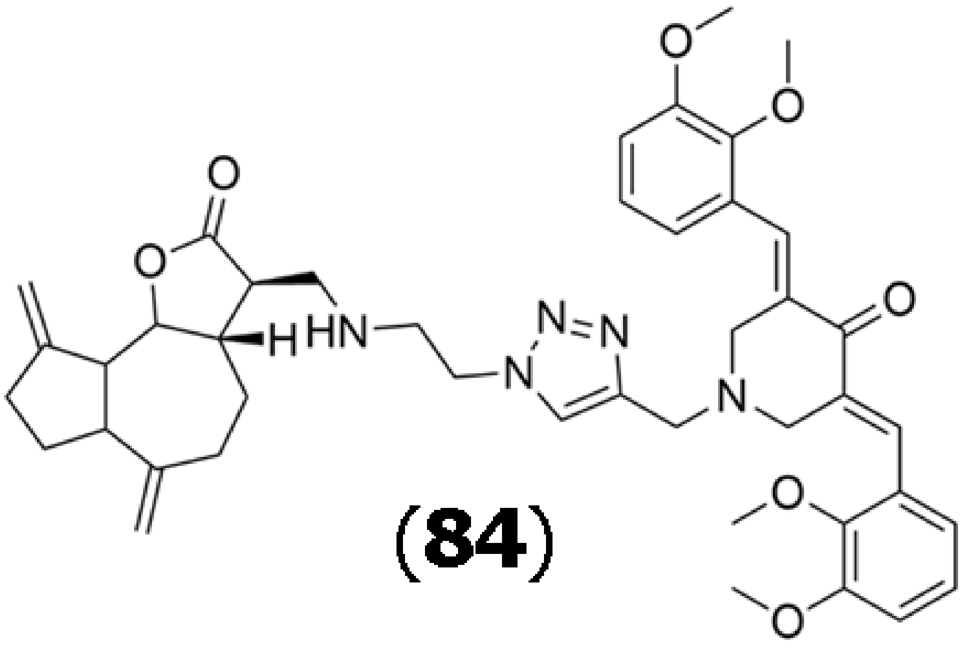

(84)

Figure S7. Chemical structures of synthesized 3,5-bis(arylidene)-4-piperidone derivatives of dehydrocostus lactone (**63**). (Adapted from Reference 63)

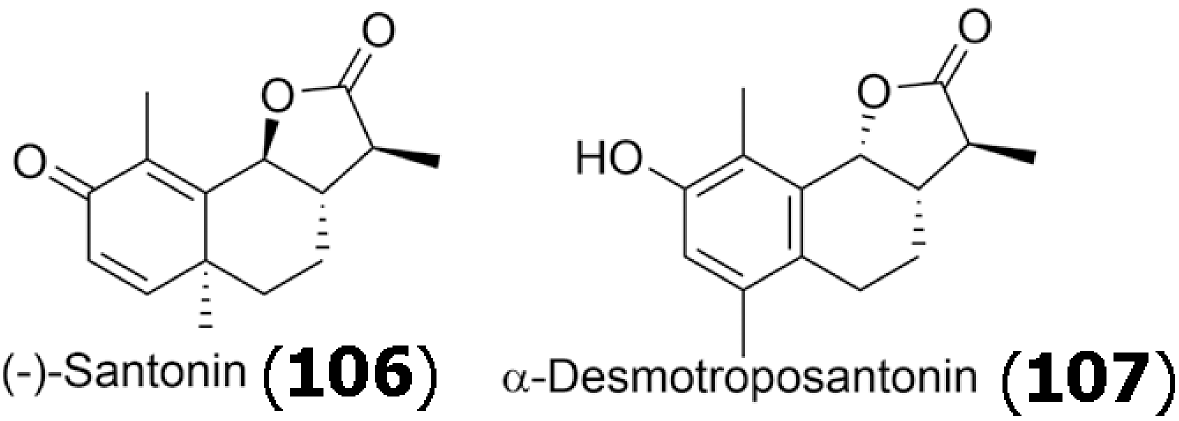

(-)-Santonin **(106)** α-Desmotroposantonin **(107)**

Figure S8. Chemical structures of (-)-santonin and α-desmotroposantonin.

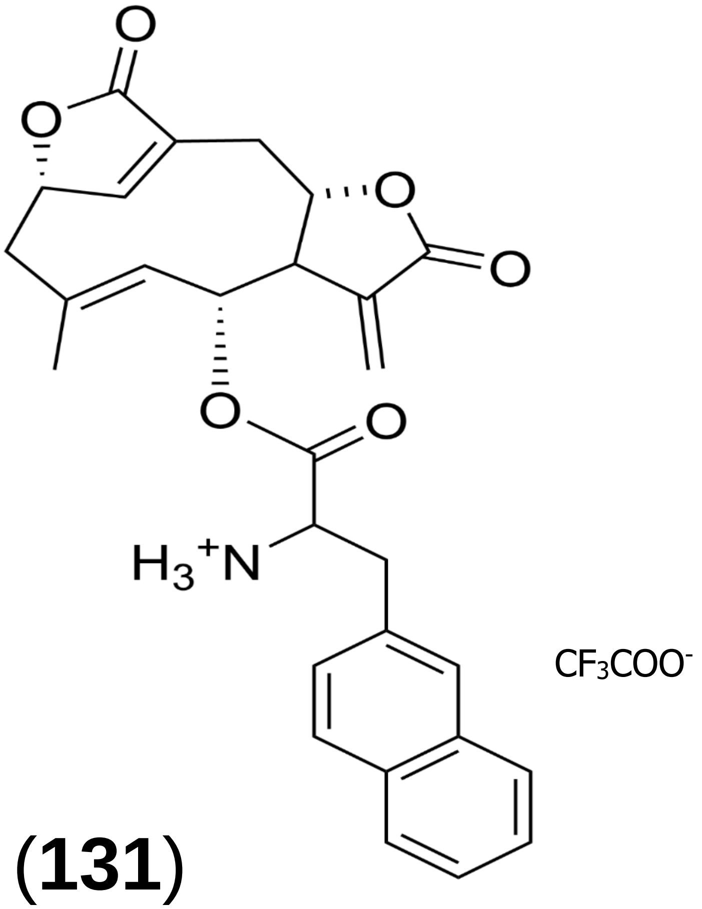

(131)

Figure S9. Amino acid ester trifluoroacetate derivative (**131**) of scaberol C (**122**) (Adapted from Reference 78).

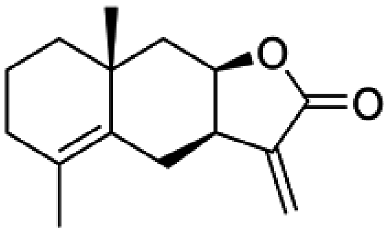

Alloalantolactone
(**139**)

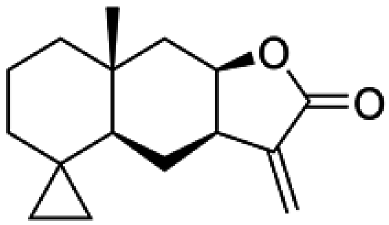

Epoxy Isoalantolactone
(**140**)

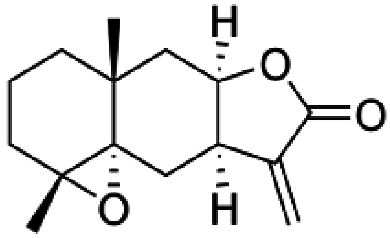

Epoxy Alloalantolactone
(**141**)

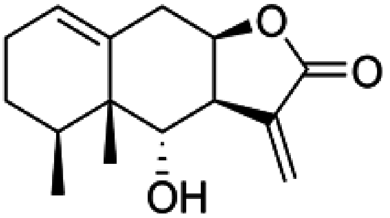

6-Hydroxyxanthanodiene
(**142**)

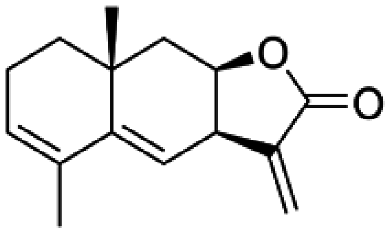

Allanthodiene
(**143**)

Figure S10. Sesquiterpene structures (**139-143**) that have been utilized for the synthesis of chemotherapeutic drugs.

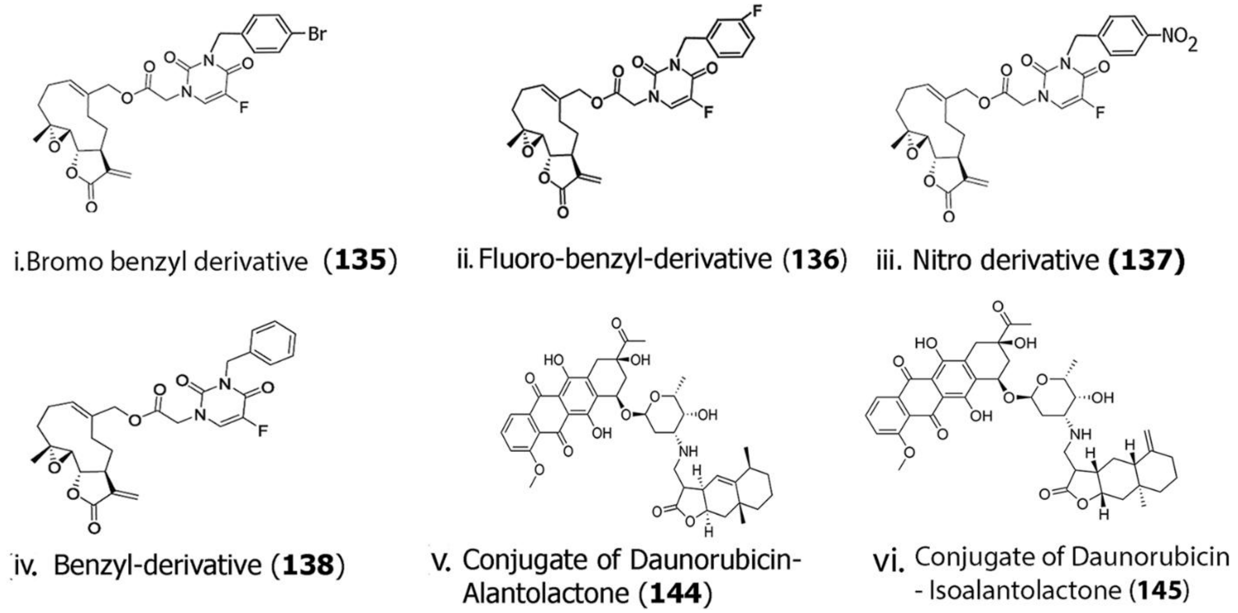

Figure S11. Chemical structures of chemotherapeutic drug-SL conjugates (i-iv) show some of the 5-fluorouracil conjugates with parthenolide (**135-138**); (v and vi) show alantolactone and isoalantolactone conjugates with daunorubicin (**144** and **145**)

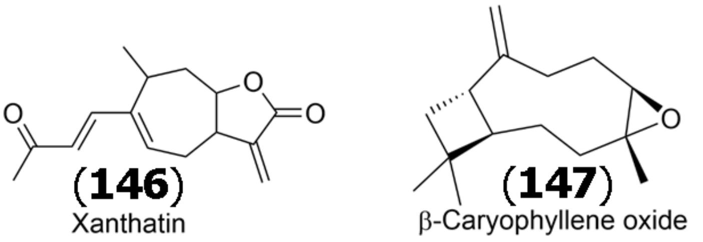

Figure S12. Chemical structures of xanthatin (**146**) and beta-caryophyllene oxide (**147**)

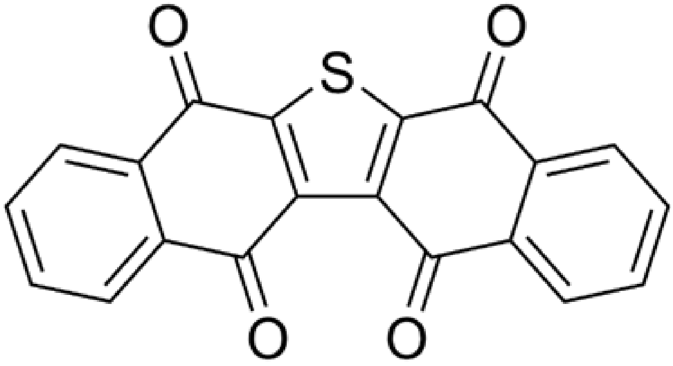

Seriniquinone
(**165**)

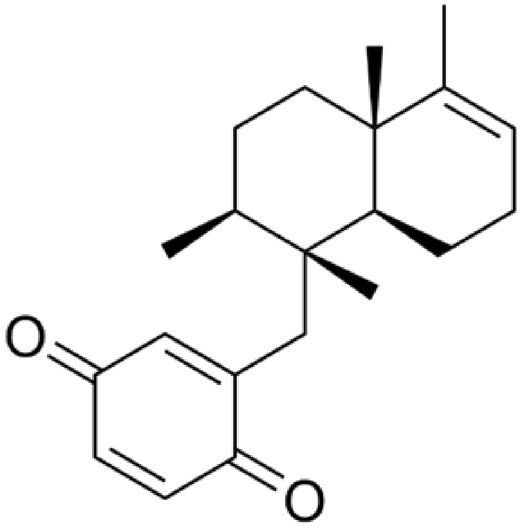

Avarone
(**166**)

Figure S13. Chemical structures of additional sesquiterpene quinones seriniquinone (**165**) and avarone (**166**).

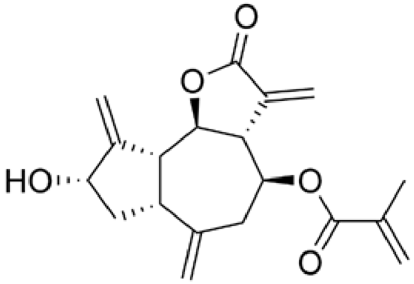

Aguerin B **(167)**

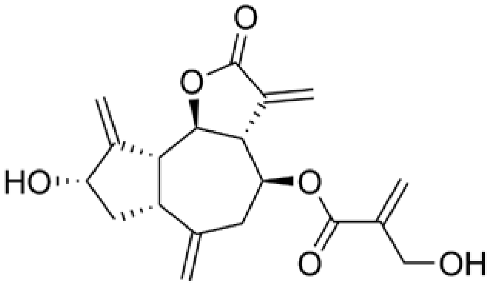

Cynaropicrin **(168)**

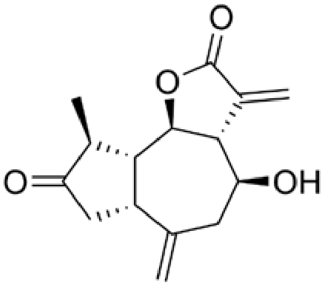

Grosheimin **(169)**

Figure S14. Chemical structures of guaiane-type sesquiterpene lactones aguerin (**167**); cynaropicrin (**168**) and grosheimin (**169**) used in nanoformulations with lithocholic acid.

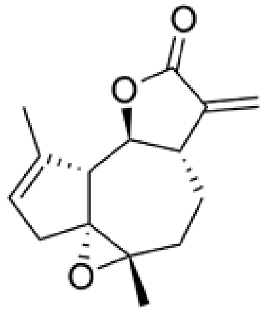

(170)
Arglabin

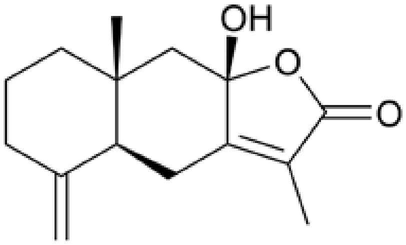

(171)
Atracytenolide

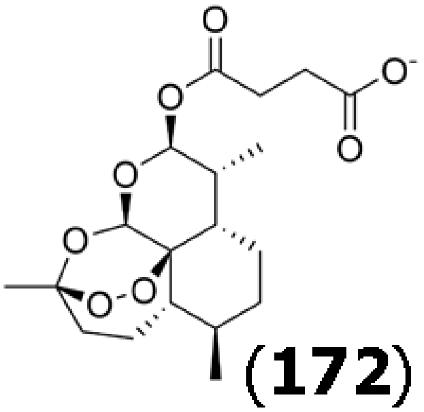

(172)
Artesunate

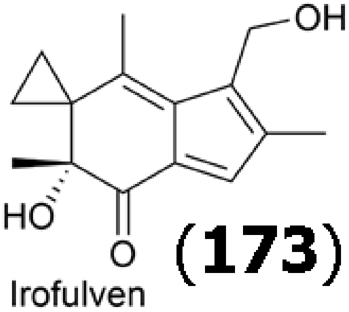

(173)
Irofulven

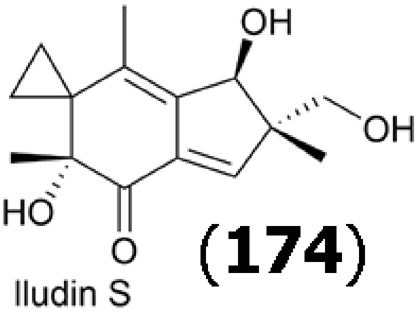

(174)
Iludin S

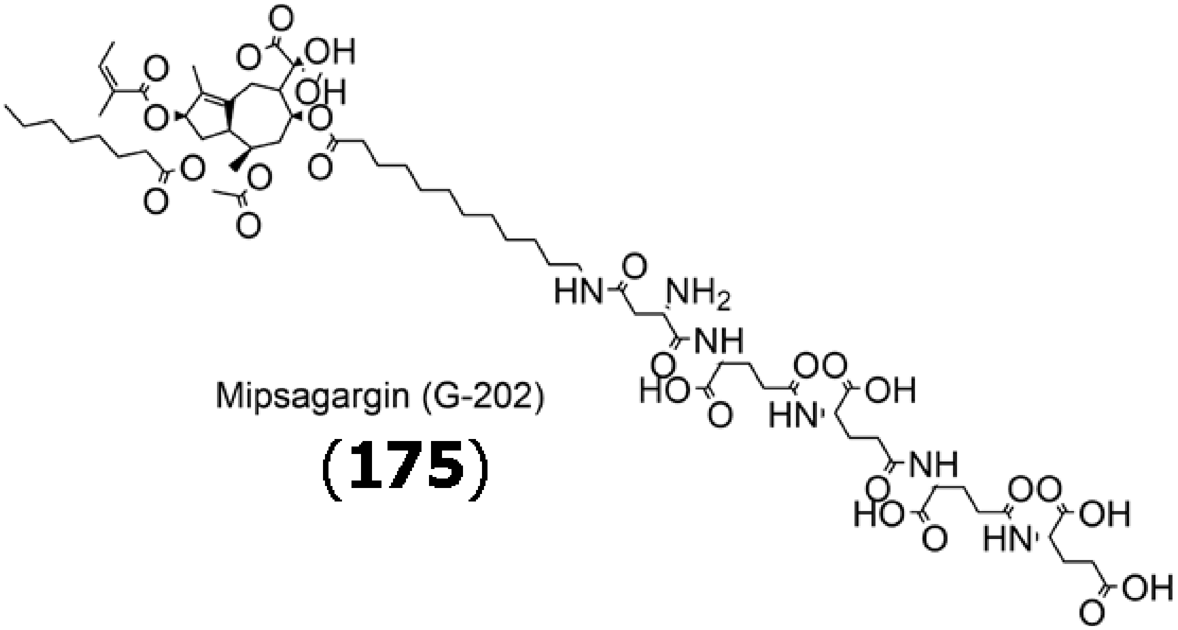

Mipsagargin (G-202)
(175)

Figure S15. Chemical structures of sesquiterpenes and some of their modified derivatives that have undergone clinical trials.

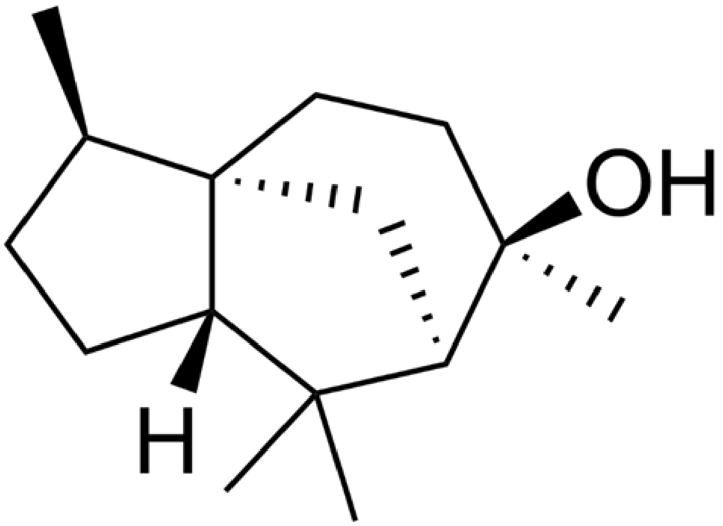

Cedrol (**186**)

Figure S16. Chemical structure of sesquiterpene alcohol Cedrol (**186**).

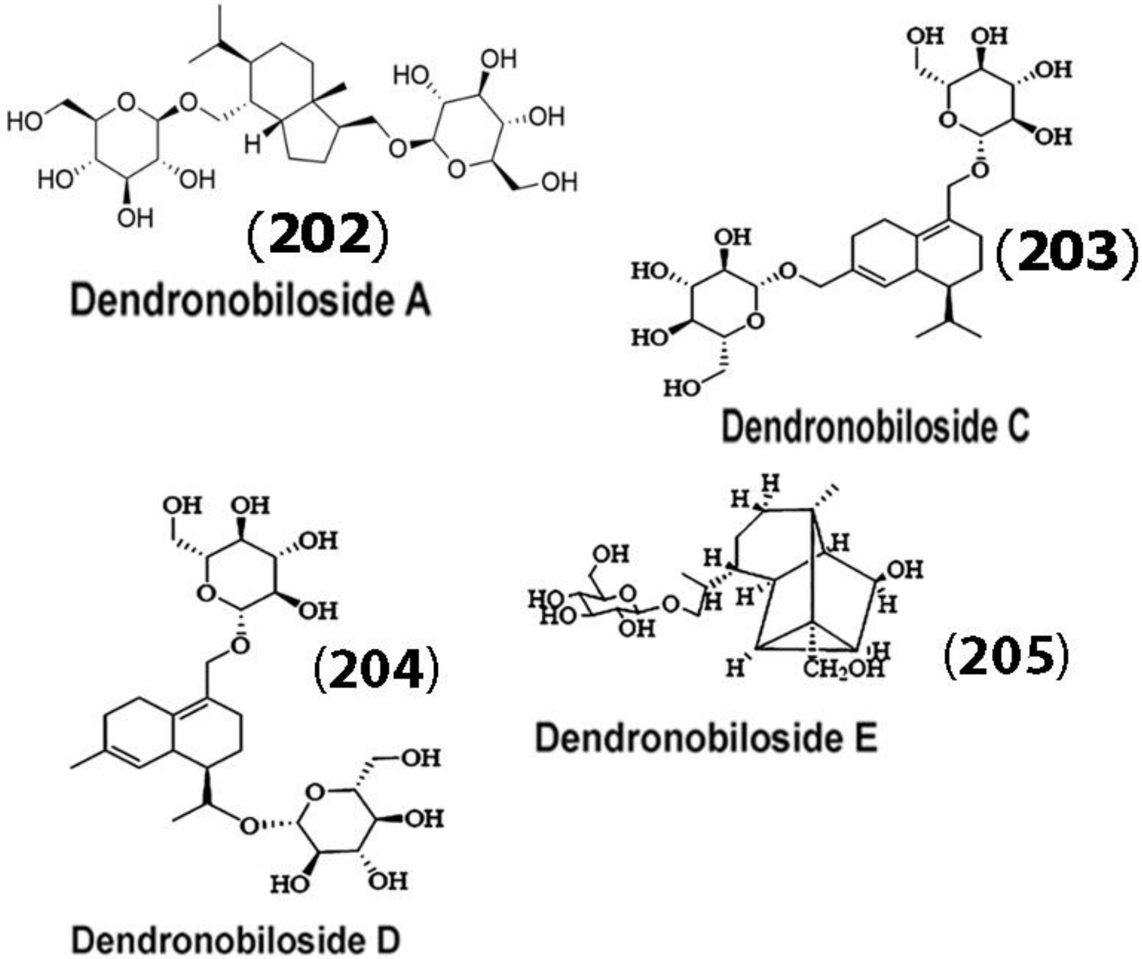

Figure S17. Chemical structures of dendronobilosides A, C, D and E (compounds (**202**) through (**205**))

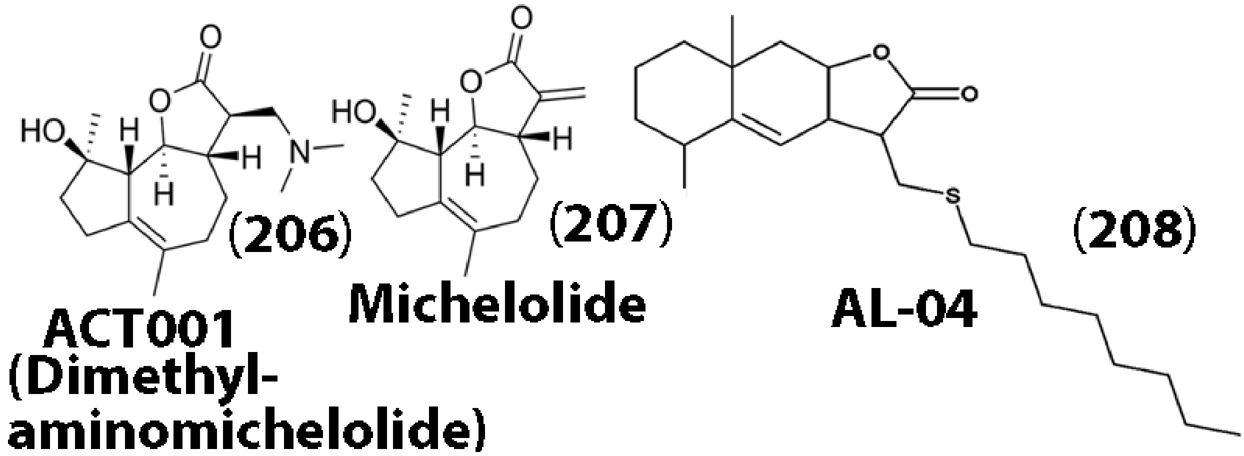

Figure S18. Chemical structure of michelolide derivative ACT001 (**206**); micheliolide (**207**) and thiolated derivative of alantolactone (AL-04) (**208**).
